# Treatment of breast cancer with autophagy inhibitory microRNAs carried by AGO2-conjugated nanoparticles

**DOI:** 10.1186/s12951-020-00615-4

**Published:** 2020-04-28

**Authors:** Ozlem Unal, Yunus Akkoc, Muhammed Kocak, Esra Nalbat, Asiye Isin Dogan-Ekici, Havva Yagci Acar, Devrim Gozuacik

**Affiliations:** 1 Koc University, Graduate School of Material Science and Engineering, Rumelifeneri Yolu Sarıyer, 34450 Istanbul, Turkey; 2 Sabanci University Nanotechnology Research and Application Center (SUNUM), Department of Biotechnology, Tuzla, 34956 Istanbul, Turkey; 3 Acıbadem Mehmet Ali Aydınlar University, School of Medicine Department of Pathology, Ataşehir, 34755 Istanbul, Turkey; 4 Koc University, Department of Chemistry, Rumelifeneri Yolu Sarıyer, 34450 Istanbul, Turkey; 5 Koc University Surface Science and Technology Center (KUYTAM), Rumelifeneri Yolu Sarıyer, 34450 Istanbul, Turkey; 6 Koç University, School of Medicine, Koç University Research Center for Translational Medicine (KUTTAM), Topkapı, 34010 Istanbul, Turkey

**Keywords:** SPION, Theranostic nanoparticle, Cancer treatment, Gene therapy, MicroRNA, MIR376B, Autophagy

## Abstract

Nanoparticle based gene delivery systems holds great promise. Superparamagnetic iron oxide nanoparticles (SPIONs) are being heavily investigated due to good biocompatibility and added diagnostic potential, rendering such nanoparticles theranostic. Yet, commonly used cationic coatings for efficient delivery of such anionic cargos, results in significant toxicity limiting translation of the technology to the clinic. Here, we describe a highly biocompatible, small and non-cationic SPION-based theranostic nanoparticles as novel gene therapy agents. We propose for the first-time, the usage of the microRNA machinery RISC complex component Argonaute 2 (AGO2) protein as a microRNA stabilizing agent and a delivery vehicle. In this study, AGO2 protein-conjugated, anti-HER2 antibody-linked and fluorophore-tagged SPION nanoparticles were developed (SP-AH nanoparticles) and used as a carrier for an autophagy inhibitory microRNA, *MIR376B*. These functionalized nanoparticles selectively delivered an effective amount of the microRNA into HER2-positive breast cancer cell lines in vitro and in a xenograft nude mice model of breast cancer in vivo, and successfully blocked autophagy. Furthermore, combination of the chemotherapy agent cisplatin with *MIR376B*-loaded SP-AH nanoparticles increased the efficacy of the anti-cancer treatment both in vitro in cells and in vivo in the nude mice. Therefore, we propose that AGO2 protein conjugated SPIONs are a new class of theranostic nanoparticles and can be efficiently used as innovative, non-cationic, non-toxic gene therapy tools for targeted therapy of cancer.

## Introduction

Most human diseases are caused by abnormalities and malfunction of genes and proteins. Hence, gene therapy of diseases has been considered as an alternative treatment approach in the last decades. A major limitation in gene therapy is related to the choice of gene delivery vehicle. Viral vectors used as a general vehicle in many of the studies suffer from a number of adverse reactions and side-effects, including immune reactions against carrier viruses and mutagenesis related to the viral genomic integrations etc. [1–3]. Nanoparticles emerge as alternative and safer gene delivery vehicle candidates. A wide range of cationic polymers, i.e. polyethylene imine (PEI) [4], cationic dendrimers [5], polymeric nanoparticles, i.e. Chitosan/poly (lactide-*co*-glycolide) (PLGA) [6], lipid derivatives [7], collagen derivatives [8], and inorganic nanoparticles with cationic coatings such as silica nanoparticles [9], gold nanoparticles (Au NPs) [10] were tested both in in vitro and in vivo models of diseases as alternative gene delivery vehicles. Cationic polymers offer a large number of functionalities which allow high loading capacity, co-delivery of other therapeutic agents, attachment of targeting ligands. However, these systems are generally toxic to cells and tissues, limiting their dosage and applications. Moreover, they lack the imaging modality which would provide tremendous advantages in the monitoring of delivery, disease progress and therapy. From this perspective, designing inorganic nanoparticles with diagnostic modality as gene delivery vehicles offers an immense potential.

Superparamagnetic iron oxide (Fe_2_O_3_) nanoparticles (SPIONs, SP) were widely used in the core of various gene delivery systems. SPIONs are biocompatible and non-toxic and they have been commonly used as FDA-approved magnetic resonance imaging contrast agents [11]. They can also be manipulated, targeted and heated using an external magnetic field, making them ideal candidates for theranostic medical applications, including gene therapy [12–17].

Suitable coating materials over the crystalline iron oxide core are required to allow binding to nucleic acids. Such coatings need to be cationic, e.g. PEI [18], chitosan [19], PAMAM dendrimer [20], since the nucleic acid cargo is anionic. Toxicity of the cationic polymers, non-specific uptake and immune reactions, might be observed due to positive charges on these delivery agents and limit their successful usage in the clinic [21, 22]. Furthermore, improvement of selectivity of the delivery vehicle is critical in order to enhance the therapeutic effect while reducing the side effects. Targeting may be achieved through attachment of ligands, such as peptides, proteins or small molecules that selectively recognize target cells and improve internalization. Moreover, nanoparticles should be small enough to escape from the reticuloendothelial system (RES, including mononuclear phagocyte system of liver and spleen). Hence, there is an urgent need for non-toxic, small, target specific and theranostic nanoparticles, allowing efficient nucleic acid binding and delivery as well as medical imaging simultaneously.

Various nucleic acid molecules, e.g. DNA, RNA, modified nucleotides, are being utilized in gene therapy attempts. MicroRNAs (miRNAs) are small (18–21 nucleotide), protein non-coding, endogenous RNA molecules that regulate key biological events, including cell proliferation, differentiation and survival. MiRNAs modulate cellular levels and translation of dozens of messenger RNAs, controlling abundance of their protein products [23]. By this way, they determine cellular responses to various stimuli under physiological and pathological conditions. Indeed, dysregulation, i.e. upregulation or downregulation, of several miRNAs was observed in a wide variety of health problems, including cancer [24].

To date, more than 2000 miRNAs were described in the human genome. MicroRNA genes were found in both intergenic or intragenic gene loci. Following RNA polymerase II-dependent transcription, maturation of miRNA precursors is processed by several protein complexes that are found in the nucleus and the cytosol [25].

Resulting mature miRNA duplexes are eventually loaded onto the RNA-induced silencing complexes (RISC) in the cytosol. Argonaute (AGO) proteins are key components of the RISC. They bind to single-stranded mature microRNAs, guiding them to their target messenger RNAs (mRNAs), and leading to mRNA degradation or translation blockage [26]. Moreover, AGO proteins contribute to the stability of mature miRNA strands in RNAse containing biological environments. Indeed, cell- or vesicle-free and stable mature miRNAs were found in complex with AGO proteins in the blood circulation [27–30]. In vitro tests confirmed that AGO2 increase the stability of these small RNAs [31–35].

Recent studies showed that miRNAs tightly regulate autophagy, an evolutionarily conserved cellular stress response mechanism [25]. For example, we have previously showed that *MIR376B* blocked excessive cellular autophagy through targeting of its key components *BECN1* and *ATG4C* [36, 37]. Autophagy was shown to support survival of cells that are exposed to stressful conditions, including chemotherapy agents. Consequently, in established tumors, combination of autophagy blocking agents and chemotherapy drugs resulted in more efficient tumor elimination than single agent treatments [38–40].

Here, inspired by the stability of naturally occurring AGO protein/miRNA complexes in the blood circulation, we have designed AGO2 conjugated SPIONs as tumor targeted miRNA delivery vehicles for gene therapy of cancer. As the initial target, we have studied breast cancer as a model and developed AGO2 protein conjugated SPIONs (SP-A) and used HER2 antibodies as targeting moieties. Addition of a fluorescent tag on the antibody allowed optical detection of the particles in vitro and in vivo (SP-AH nanoparticles). SP-AH were successfully synthesized from poly(acrylic acid)-coated SPIONs with excellent colloidal stability and in small sizes. This is the first example to AGO2 protein containing nanoparticles and the first demonstration of AGO2/nanoparticle conjugate as a transfection agent.

Autophagy inhibiting *MIR376B* miRNAs were selectively delivered into HER2-positive breast cancer cell lines (SKBR3 and MDA-MB-453) by SP-AH nanoparticles. Effective inhibition of autophagic activity by nanoparticles was demonstrated in vitro in cells and in vivo xenograft model of breast cancer. Furthermore, we showed that combination of *MIR376B*-loaded SP-AH nanoparticles with chemotherapy drug cisplatin, significantly potentiated the tumor response to therapy both in vitro and in vivo. Therefore, we introduce the AGO2 protein as a powerful and stable miRNA carrier component for non-cationic, nanoparticle-based gene delivery approaches.

## Results and discussion

### Characterization of AGO2 as a miRNA stabilizer protein

Previous reports indicated that cell-free miRNAs were found in blood circulation in complex with the AGO2 protein that increased their stability [31, 32]. In order to confirm these observations, we have overexpressed the AGO2 protein, performed immunoprecipitation assays and tested the amount of bound miRNA using qPCR. AGO2 overexpression led to six times increase in the amount of protein-bound endogenous mammalian *MIR376B* levels compared to control (Additional file 1: Fig. S1a, b). In order to determine the effect of AGO2 on miRNA stability, we have incubated immunoprecipitates for 21 days at room temperature. Even after this incubation period, a significant amount of miRNA was still detected by qPCR, indicating that AGO2 protein bound miRNAs were protected from degradation (Additional file 1: Fig. S1c). These results indicated that AGO2 protein may be used as a miRNA binding and stabilizing tool in gene therapy.

### Synthesis and characterization of AGO2 conjugated and anti-HER2 labeled SPIONs (SP-AH)

**Fig. 1:**
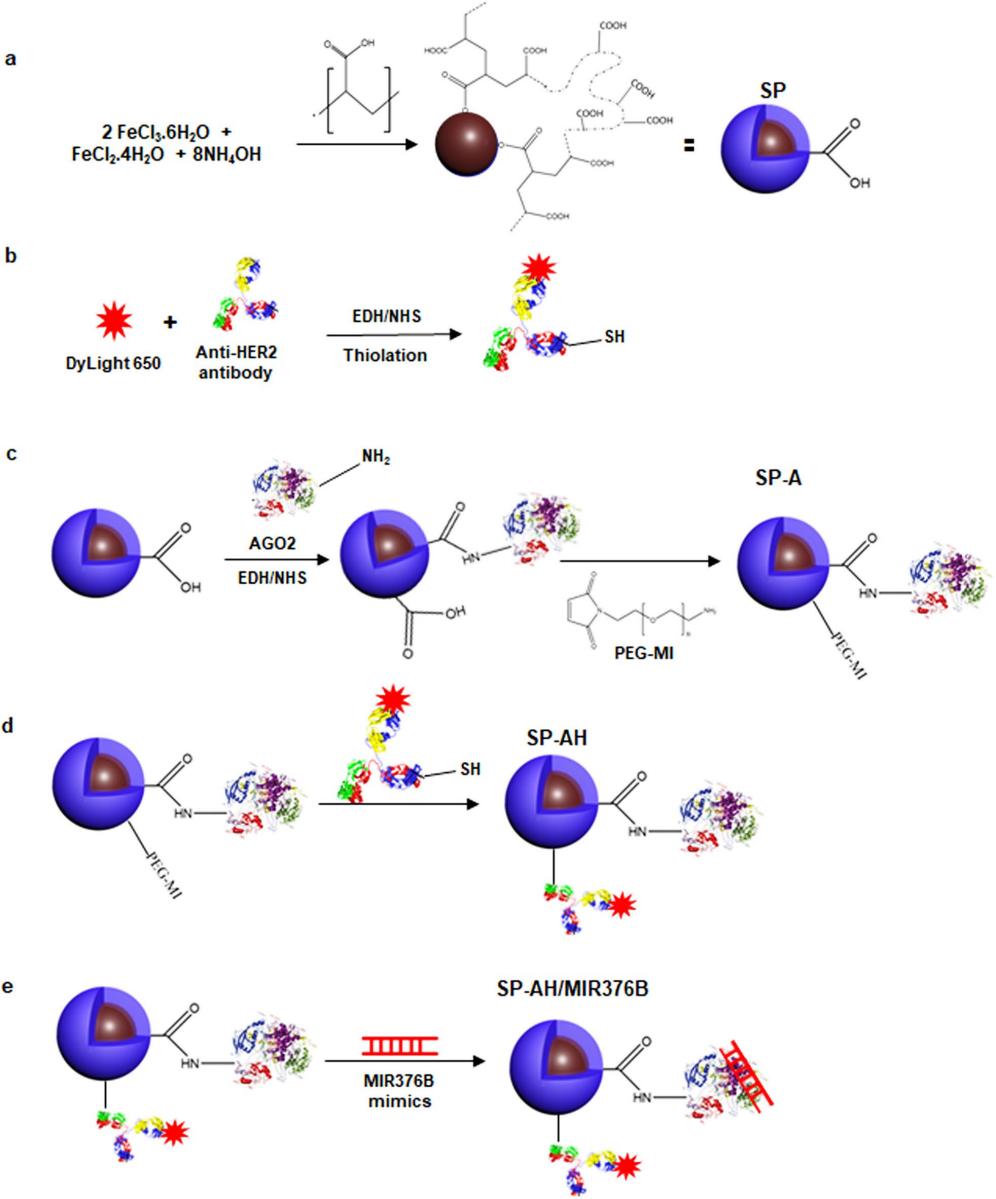
Schematic representation of SPION/AGO2/antiHer2-*MIR376B* (SP-AH/MIR376B) synthesis. **a** Synthesis of PAA-coated superparamagnetic iron oxide nanoparticles (SP). **b** Fluorescent labeling and thiolation of anti-HER2 antibody. **c** Conjugation of recombinant AGO2 protein to PAA coated SPIONs via amide bond generating SP-A and its functionalization with maleimide terminated PEG linker (39.7 Å). **d** Conjugation of dye labeled and thiolated anti-HER2 antibody to maleimide functional SP-A generating SP-AH. **e** Loading of *MIR376B* mimic on SPION/AGO2/antiHer2 (SP-AH) NPs

**Fig. 2:**
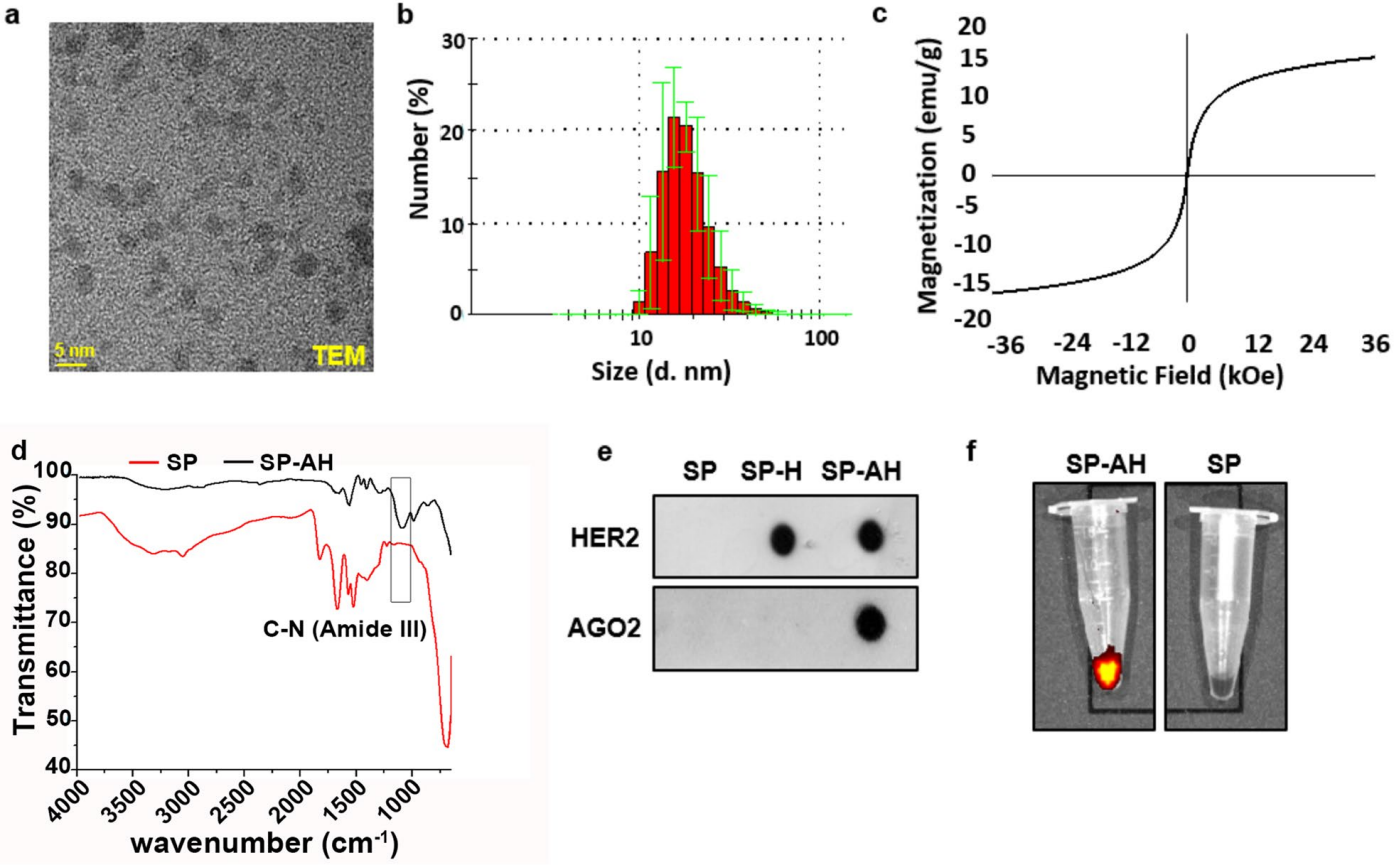
Characterization of synthesized nanoparticles. **a** TEM micrograph of SP (Scale bar: 5 nm). **b** Number average hydrodynamic size distribution measured by DLS. **c** Magnetization curve of SP as a function of magnetic field at 300 K. **d** FTIR spectra of SP, SP-A and SP-AH. **e** Dot blot analysis of SP, SP-H and SP-AH. 150 µg from each nanoparticle was loaded on the dot blot nitrocellulose membrane. **f** Detection of the optical signal from SP-AH nanoparticles using IVIS imaging system

Since synthesis of small colloidal SPIONs requires relatively high reaction temperature and basic condition as well as large excess of the coating molecule, first highly functional and stable poly(acrylic acid) coated SPIONs (SP) were prepared and then conjugated with the recombinant full length AGO2 protein as a nucleic acid binding component (Fig. 1). Poly(acrylic acid) coated SPIONs (SP) were synthesized via an aqueous co-precipitation of ferric and ferrous salts under alkaline conditions (Fig. 1a). TEM micrograph shows small crystalline cores of nearly 4 nm without a noticeable aggregation (Fig. 2a and Additional file 1: Fig. S7a). EDX analysis further demonstrated Fe and O composition of SPIONs (Additional file 1: Fig. S7a). Small size and colloidal stability of SP were also confirmed with 17 nm hydrodynamic size (number average) measured by DLS and strong negative surface charge, − 19 meV (Fig. 2b and Additional file 1: Fig. S2a). XRD diffraction patterns of SP nanoparticles belong to the iron oxide spinel structure of magnetite (Fe_3_O_4_) (Fig. 2b). Broadening in the peaks is attributed to the nanometer size of powder analyte. SP showed no hysteresis when subjected to magnetization–demagnetization cycle indicating the superparamagnetic nature of these nanoparticles (Fig. 2c). Thermogravimetric analysis (TGA) of SP showed a two-stage degradation similar to pure PAA and the PAA content of SP was determined as 36% by weight (Fig. 2c). Presence of the poly(acrylic acid) (PAA) coating on SPION core was confirmed with the strong symmetric stretching band of carbonyl (1570 cm^−1^) and broad band of hydroxyl groups (2800–3700 cm^−1^) representing the –COOH functionality of PAA in the FTIR spectrum of the SP (Fig. 2d and Additional file 1: Fig. S2d). In all later conjugation reactions performed on these SP, this PAA content was used as the basis of stoichiometry.

Recombinant full length AGO2 protein was covalently attached to –COOH groups of SP via direct amide bond using the two step EDC/NHS chemistry producing the AGO2-conjugated SPIONs (SP-A) (Fig. 1c). AGO2 content of the SP-A was calculated spectroscopically using the Bradford Assay (Additional file 1: Fig. S3a), as 5.5 µg AGO2 per mg of nanoparticles.

In order to target nanoparticles to HER2-positive breast cancer cells, these nanoparticles were tagged with fluorescent dye labeled anti-HER2 antibody using a 39.7 Å PEG spacer. This strategy aims to protrude the anti-HER2 from the bulk of the nanoparticle coating (PAA and AGO2) for effective interaction of anti-HER2 with the cell surface receptor. First, maleimide functional SP prepared via direct amidation of SP with maleimide-PEG-NH_2_. For the conjugation, the anti-HER2 antibody was thiolated using the Traut’s reagent. Nearly 55 sulfhydryl units were calculated per anti-HER2 with the Ellman’s test. Then, thiolated anti-HER2 was coupled with the maleimide functional SP via thiol-ene chemistry (Fig. 1d). For the optical tracking of nanoparticles, and confirmation of anti-HER2 conjugation to SP-A, antibody was labelled with a far-red fluorophore (Dylight650) (Fig. 1b). This strategy also placed the fluorescent tag away from the SPION core which is a strong absorber in the visible region, maximizing the intensity of the dye for optical detection. The fluorophore tag and hence the amount of anti-HER2 antibody on SP-AH nanoparticles were quantified from the luminescence intensity of the dye at 672 nm as nearly 0.8 mol of dye per mole of antibody and 24 anti-HER2 per particle (Additional file 1: Fig. S3b, c). Protein conjugations to SP was confirmed by the appearance of amide peaks in FTIR (Fig. 2d) and specific Dot-blot (Fig. 2e) analysis. Ability to detect the fluorescently labeled SP-AH by IVIS imaging system was confirmed before the animal studies and compared with non-fluorescent SP (Fig. 2f).

SP conjugated only with anti-HER2 antibody (SP-H) or only with the fluorophore (SP-F) as well as fluorophore-labeled AGO2 conjugated nanoparticles (SP-AF) were also prepared as control nanoparticles to be used in targeting and miRNA binding assays. The fluorophore on SP-F and SP-AF nanoparticles were determined from the fluorescence intensity of the dye at 672 nm (Additional file 1: Fig. S3d, e). Particle properties including the hydrodynamic size (nm), poly dispersity indexes (PDI), zeta potential (mV) as well as composition of each nanoparticle are summarized in Table 1. One very important point to notice here is the small size of the target particle SP-AH which is 50 nm and zeta potential which is − 24 mV. The synthetic approach was designed to guarantee the small size to prevent fast clearance from the blood stream. Commonly used gene delivery systems are generally cationic, limiting their biocompatibility and increasing their toxicity. Negative surface charge of AGO2-conjugated nanoparticles is another novelty of this nanoparticle designed for gene delivery.

**Table 1:** Physical characterization and composition of SPION nanoparticles

Sample	Acronym	Z-average HDR* (nm)	PDI	Zeta Pot. (mV)	AGO2 μg/ml	HER2 μg/ml
SPION/PAA	SP	14	0.40	− 19	0	0
SPION/PAA/AGO2	SP-A	20	0.43	− 23	30	0
SPION/PAA/HER2	SP-H	30	0.59	− 21	0	91.5
SPION/PAA/AGO2/HER2	SP-AH	49	0.23	− 24	30	41
*SPION/PAA	SP-F	43	0.29	− 23	0	0
*SPION/PAA/AGO2	SP-AF	70	0.40	− 20	95	0

*Fluorophore conjugated nanoparticles

### Investigation of biocompatibility and potential side-effects of SP-AH nanoparticles

In order to confirm biocompatibility, we treated MCF-7, SKBR3 or MDA-MB-453 breast cancer cells with increasing concentrations (5–500 µg/ml; for 48 h) of SP-AH or control SP nanoparticles and evaluated cell survival in vitro. No significant difference in cell viability was observed even at the highest concentration of nanoparticles. Neither SP-AH nor SP were toxic to cells after 48 h of treatment (Additional file 1: Fig. S4). Next, SP-AH or SP nanoparticles were *i.v.* injected to mice through tail vein. No significant change in body weight was observed in nanoparticle injected mice compared to age-matched PBS injected mice after 10 or 40 days of follow-up (Additional file 1: Fig. S4b). Moreover, blood biochemistry and complete blood count showed no prominent difference between the SP-AH and SP nanoparticles versus PBS injected mice at 10 and even 40 days after the injection (Additional file 1: Tables S1 and S2). Histopathology analyses of the major organs of these mice did not reveal any abnormality (Additional file 1: Fig. S4c). In addition, the iron content of various organs, including liver, spleen and kidney, of SP-AH administered mice determined by inductively coupled plasma (ICP) showed no difference between PBS- and nanoparticle-injected mice, indicating that SP-AH nanoparticles were metabolized and did not accumulate in the tissues (Fig. 5a). Therefore, we concluded that nanoparticles used in this study are biocompatible at a cellular and organismal level both in short- and long-term tests.

### Targeting of HER2-positive breast cancer cells by SP-AH nanoparticles

**Fig. 3:**
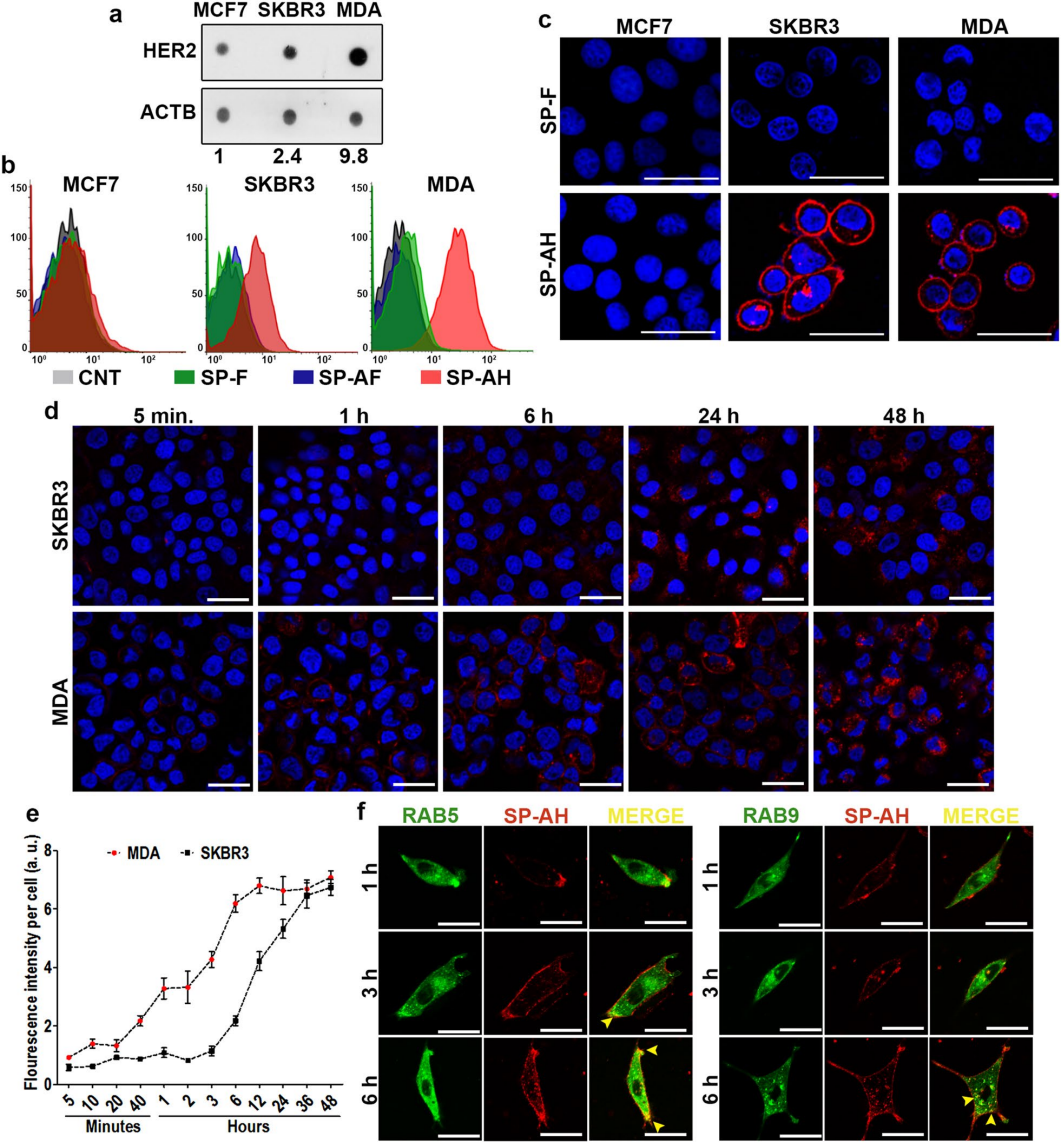
SP-AH nanoparticles targeted breast cancer cells in vitro. **a** Dot blot analysis of HER2 receptor levels of MCF7, SKBR3 and MDA-MB-453 (MDA) breast cancer cells. Actin was used as loading control (n = 3). **b** Flow cytometry (FACScan) analysis of MCF7, SKBR3 and MDA cells treated with fluorescent-labeled SP-F, SP-AF, and SP-AH nanoparticles (representative analysis was shown, n = 3). Untreated cells were used as control (CNT). **c** Confocal microscopy images of SP-F- or SP-AH-treated MCF7, SKBR3 and MDA cells. Hoechst staining was used to mark the nuclei (blue). **d** Time kinetics (5 min, 1 h (h), 6 h, 24 h and 48 h) of SP-AH nanoparticle attachment and uptake in SKBR3 or MDA cells. Blue signal, Hoechst staining of nuclei. **e** Graph depicting the ImageJ analysis of red fluorescence intensity per cell versus time in SKBR3 and MDA cells: a.u., arbitrary fluorescence signal intensity units. **f** Time-dependent (1 h, 3 h and 6 h) colocalization (merge, yellow) of SP-AH nanoparticles (red) with the early endosome markerRAB5 (green). Scale bar: 25 µm

HER2/NEU/ERBB2 is a member of the epidermal growth factor receptor family and 10–30% of breast tumors are HER2 positive [41]. We first determined the HER2 expression levels of three different breast cancer-derived cell lines. Dot blot analysis using an anti-HER2 antibody revealed that highest expression of the receptor was observed in MDA-MB-453 and SKBR3 cells, while MCF-7 cells were low expressors (Fig. 3a). Next cells were treated with PBS control or fluorophore-labeled SP-F, SP-AF or SP-AH nanoparticles and FACScan analyses were performed. While fluorescent signal obtained from SP-F and SP-AF-treated cells were at the auto-fluorescence level of control cells, there was a clear signal-shift proportional to the HER2 expression levels of cells when treated with SP-AH nanoparticles, indicating successful receptor-mediated targeting of MDA-MB-453 and SKBR3 cells with anti-HER2-conjugated SP-AH (Fig. 3b). Confocal microscopy analysis results were in line with FACScan results. SKBR3 and MDA-MB-453 cell membranes were brightly stained with SP-AH nanoparticles, yet, no signal was observed in HER2 low expressor MCF-7 cells or cells treated with SP-F nanoparticles devoid of HER2 antibody (Fig. 3c).

In order to assess receptor-mediated uptake kinetics and cellular destination of SP-AH nanoparticles, SKBR3 and MDA cells were treated with SP-AH nanoparticles and fluorescent signals were followed at various time points. Cellular targeting of nanoparticles (150 μg/ml NPs; 5 min, 1 h (h), 6 h, 24 h and 48 h) was observed after 1 h, and the total signal intensity gradually increased over time (Fig. 3d, e). Especially at early time points, MDA cells that are high expressors of the HER2 receptor showed a stronger fluorescence signal, indicative of higher nanoparticle accumulation compared to SKBR3 cells (Fig. 3e). We also checked colocalization of nanoparticles with early (RAB5) and late (RAB9) endosomal markers (Fig. 3f). We could observe colocalization with early endosomes after 3 h and late endosomes after 6 h.

These results confirm that SP-AH nanoparticles targeted HER2-positive cells and were internalized in these cells through endocytosis.

### Targeting of HER2-positive breast cancer xenograft tumors by SP-AH nanoparticles

**Fig. 4:**
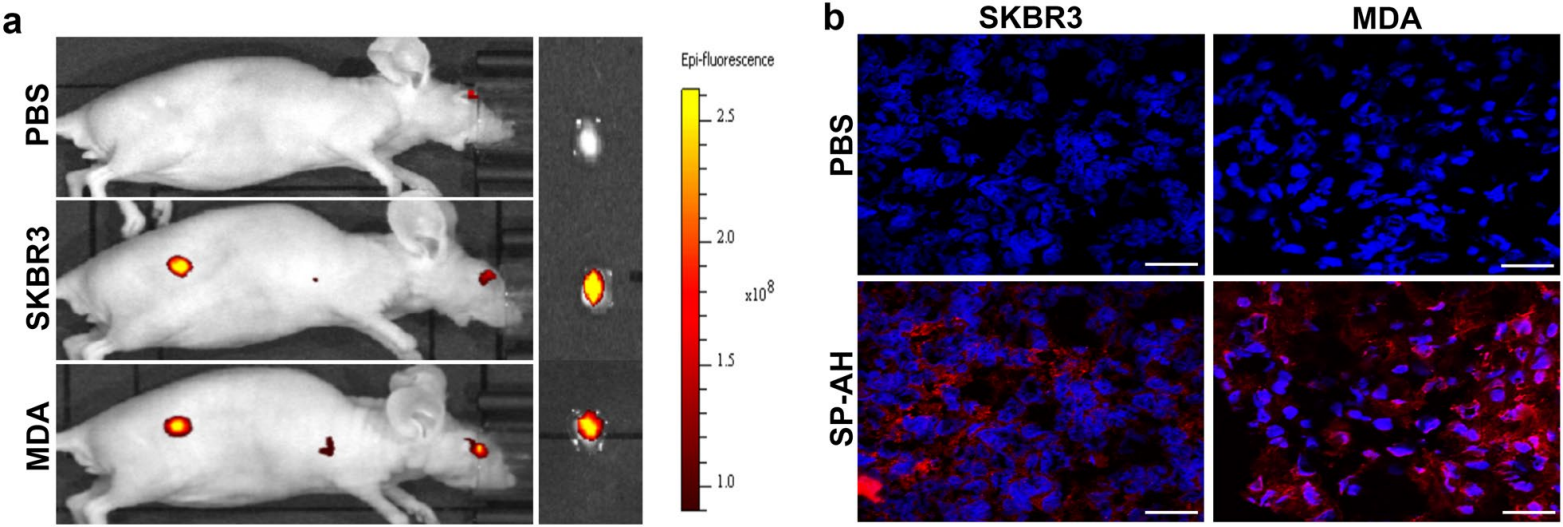
SP-AH nanoparticles targeted breast cancer cells in vivo. **a** In vivo fluorescence imaging of mice with SKBR3 or MDA xenograft breast tumors following tail vein *i.v.* injection of SP-AH nanoparticles. Control mice with SKBR3 xenograft was injected with PBS only. Ex-vivo images of the same tumors were also shown. The color scale indicates epifluorescence intensity. **b** Fluorescence images of tissue sections of tumors from PBS- or SP-AH-injected mice. Red fluorescent signal, SP-AH particles. Blue signal, Hoechst staining of nuclei. Scale bar: 50 µm

To validate targeting of HER2-positive breast tumors with SP-AH nanoparticles in vivo in mice, we created xenograft tumors of SKBR3 or MDA-MB-453 cells. When the tumors reached around 50 mm^3^, 1 month after tumor cell subcutaneous xenograft, mice were *i.v*. injected with PBS or SP-AH nanoparticles. Whole body analysis of mice under an IVIS fluorescence imaging system showed that SP-AH nanoparticles specifically and selectively accumulated in xenograft tumors but not in other organs or tissues (Fig. 4a and Additional file 1: Fig. S5b). Confocal microscopy analysis of sections of tumor specimen further confirmed tumor targeting by SP-AH nanoparticles (Fig. 4b). Moreover, ICP analysis of tumor iron content provided another proof that iron-oxide nanoparticles selectively accumulated in SP-AH-injected tumors compared to PBS-injected controls (Additional file 1: Fig. S5a).

These results demonstrate that SP-AH nanoparticles successfully and selectively targeted HER2-positive tumors in mice. Importantly, SP-AH nanoparticles were not detected in other tissues and organs.

### SP-AH-mediated delivery of *MIR376B* and suppression of autophagy in vitro

**Fig. 5:**
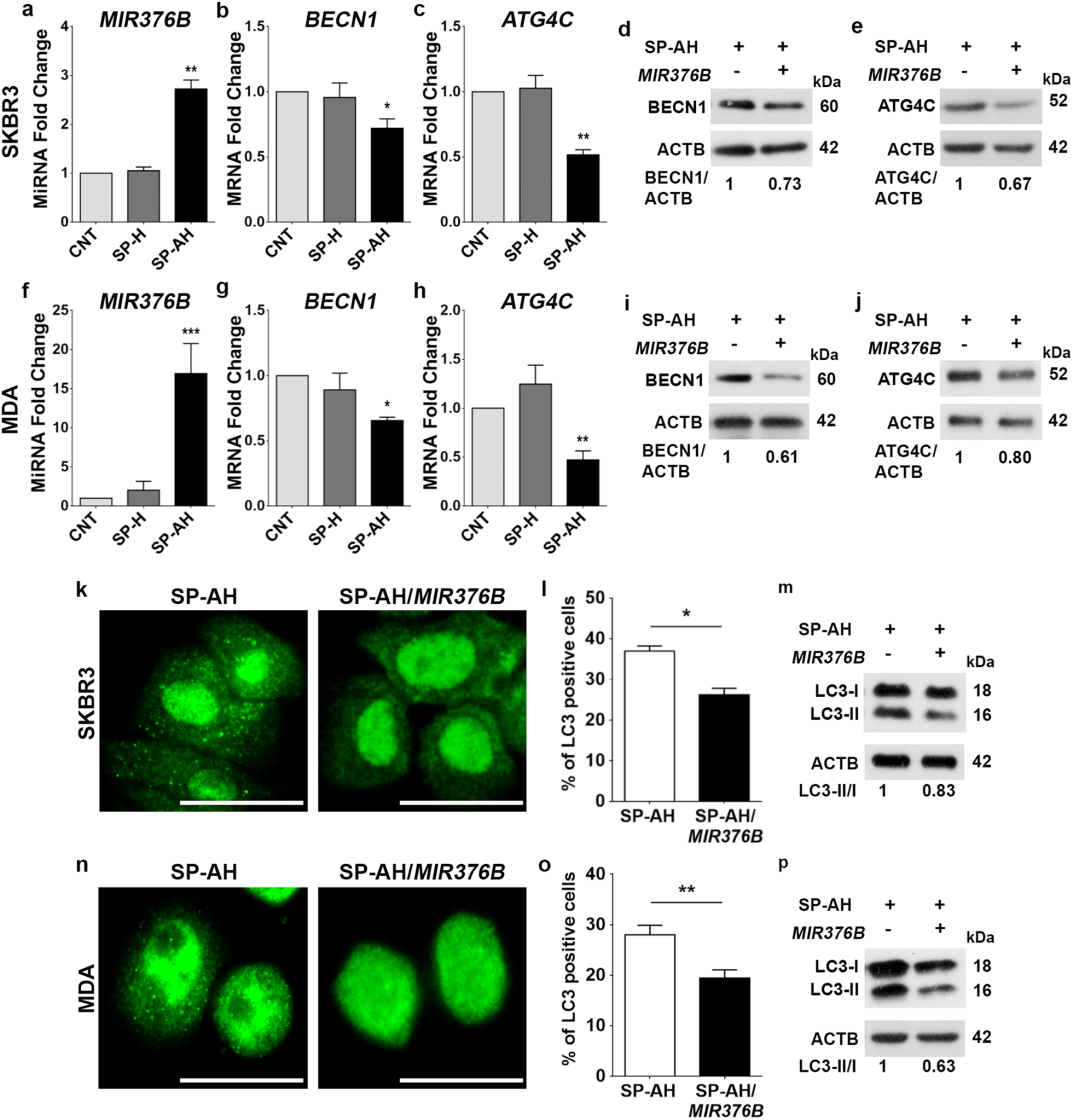
SP-AH-mediated delivery of *MIR376B* into breast cancer cells blocked autophagy. **a** Quantitative PCR (qPCR) analysis of *MIR376B* levels in control (CNT, *MIR376B* only)-, SP-H plus *MIR376B*(SP-H/*MIR376B*)- or SP-AH plus *MIR376B*(SP-AH/*MIR376B*)-treated SKBR3 cells (mean ± SD of independent experiments, n = 5, **p < 0.03). *a.u.* arbitrary units. **b** qPCR analysis of *BECN1* mRNA levels in SKBR3 cells treated as in (**a**) (n = 5, *p < 0.05). **c** qPCR analysis of *ATG4C* mRNA levels in SKBR3 cells treated as in (**a**) (n = 5, **p < 0.03). **d** Immunoblot analysis of BECN1 in the extracts from SP-AH- or SP-AH/*MIR376B*-treated SKBR3 cells. ACTB was used as the loading control. BECN1/ACTB band intensity ratios were marked. **e** Immunoblot analysis of ATG4C in the extracts from SP-AH- or SP-AH/*MIR376B*-treated SKBR3 cells. ACTB was used as loading control. ATG4C/ACTB band intensity ratios were marked. **f** qPCR analysis of *MIR376B* levels in CNT-, SP-H/*MIR376B*- or SP-AH/*MIR376B*-treated MDA cells (n = 5, ***p < 0.01). **g** qPCR analysis of *BECN1* in MDA cells treated as in (**f**) (n = 5, *p < 0.05). **h** qPCR analysis of *ATG4C* in MDA cells treated as in (**f**) (n = 5, **p < 0.03). **i** Immunoblot analysis of BECN1 in the extracts from SP-AH- or SP-AH/*MIR376B*-treated MDA cells. **j** Immunoblot analysis of ATG4C in the extracts from SP-AH- or SP-AH/*MIR376B*-treated MDA cells. **k** Fluorescence microscopy images of SP-AH- or SP-AH/*MIR376B*-treated SKBR3 cells that were immunostained with an anti-LC3 antibody. **l** Graph showing the quantification of LC3 dot positivity in SKBR3 cells (n = 3, *p < 0.05). **m** Immunoblot analysis of LC3 in the extracts from SP-AH- or SP-AH/*MIR376B*-treated SKBR3 cells. LC3-II/I band intensity ratios were marked. **n** Fluorescence microscopy images of SP-AH- or SP-AH/*MIR376B*-treated MDA cells that were immunostained with an anti-LC3 antibody. **o** Graph showing the quantification of LC3 dot positivity in MDA cells (n = 3, **p < 0.03). **p** Immunoblot analysis of LC3 in the extracts from SP-AH- or SP-AH/*MIR376B*-treated MDA cells. LC3-II/I band intensity ratios were marked. Scale bar: 25 µm

We have previously characterized *MIR376B* as an autophagy inhibitory microRNA. We showed that *MIR376B* directly targeted key autophagy genes *BECN1* and *ATG4C* and limited uncontrolled autophagy activation under cellular stress conditions creating a gas-and-break model of autophagy control [36, 37]. In fact, autophagy seems to act as a chemotherapy resistance mechanism, and several independent studies reported that combination of autophagy inhibitors with chemotherapy increased the efficacy of treatment [42–44]. Hence, inhibition of autophagy by *MIR376B* should potentiate chemotherapy-induced cell death. In an effort of using SP-AH as a miRNA delivery agent, first the conditions of loading synthetic miRNA mimics onto SP-AH nanoparticles were optimized and then the cellular outcomes were determined. *MIR376B* levels were increased by several folds following their SP-AH-mediated delivery into SKBR3 (Fig. 5a) and MDA (Fig. 5f) cells. SP-H nanoparticles that do not contain AGO2 protein were also incubated under similar conditions with the miRNA mimics, but no significant increase was observed in *MIR376B* levels in cells treated with these particles, confirming that AGO2 is the key component of the designed nanoparticle for miRNA delivery. Under these conditions, expression of *MIR376B* targets *BECN1* and *ATG4C* were also analyzed. Messenger RNA (mRNA) levels of *BECN1* (Fig. 5b, g) and *ATG4C* (Fig. 5c, h) were decreased both in SKBR3 (Fig. 5b, c) and MDA (Fig. 5g, h) cells that were treated with *MIR376B*-loaded SP-AH particles (SP-AH/*MIR376B*) compared to controls, but not with unloaded-SP-AH nanoparticles (Additional file 1: Fig. S6a, b).Furthermore, a decrease in target protein levels were observed in SKBR3 (Fig. 5d, e) and MDA cells (Fig. 5i, j) that were treated with SP-AH/*MIR376B* particles but not with SP-AH alone.

Next, autophagic activity in cells were determined using MAP1LC3B (shortly LC3) as an autophagy marker. In fact, autophagy-related lipidation of free LC3-I form of the protein leads to the formation of autophagosome-incorporated LC3-II form, resulting in a punctuate cytosolic staining pattern. Lipidation also changes migration of the protein in SDS-PAGE protein gels (18 kDa LC3-I versus 16 kDa LC3-II). LC3 dot counts and LC3-II/I ratio analysis are commonly used as autophagy detection tests. SKBR3 or MDA cells were treated with SP-AH nanoparticles with or without *MIR376B* loading, followed by autophagy assessment. SP-AH/*MIR376B* significantly inhibited LC3 dot-formation and decreased LC3-II/I ratios in SKBR3 (Fig. 5k–m) and MDA (Fig. 5n–p) cells compared to miRNA-free SP-AH nanoparticles. Hence, we conclude that, AGO2 protein containing and *MIR376B*-loaded SP-AH particles are able to efficiently downregulate autophagy-related targets of the miRNA and inhibit autophagic activity in breast cancer cells.

### SP-AH-mediated delivery of *MIR376B* and suppression of autophagy in a xenograft tumor model of breast cancer

In order to explore nucleic acid delivery potential of SP-AH nanoparticles in vivo in mice, we created a xenograft tumor model of human breast cancer. For that purpose, MDA cells were subcutaneously injected to nude mice and when tumors reached 50 mm^3^ in about 30 days, *MIR376B*-loaded SP-AH nanoparticles were administered *i.v.* through the tail vein. Six days later, tumor samples were collected and analyzed. First, miRNA delivery to tumors was confirmed by qPCR.

**Fig. 6:**
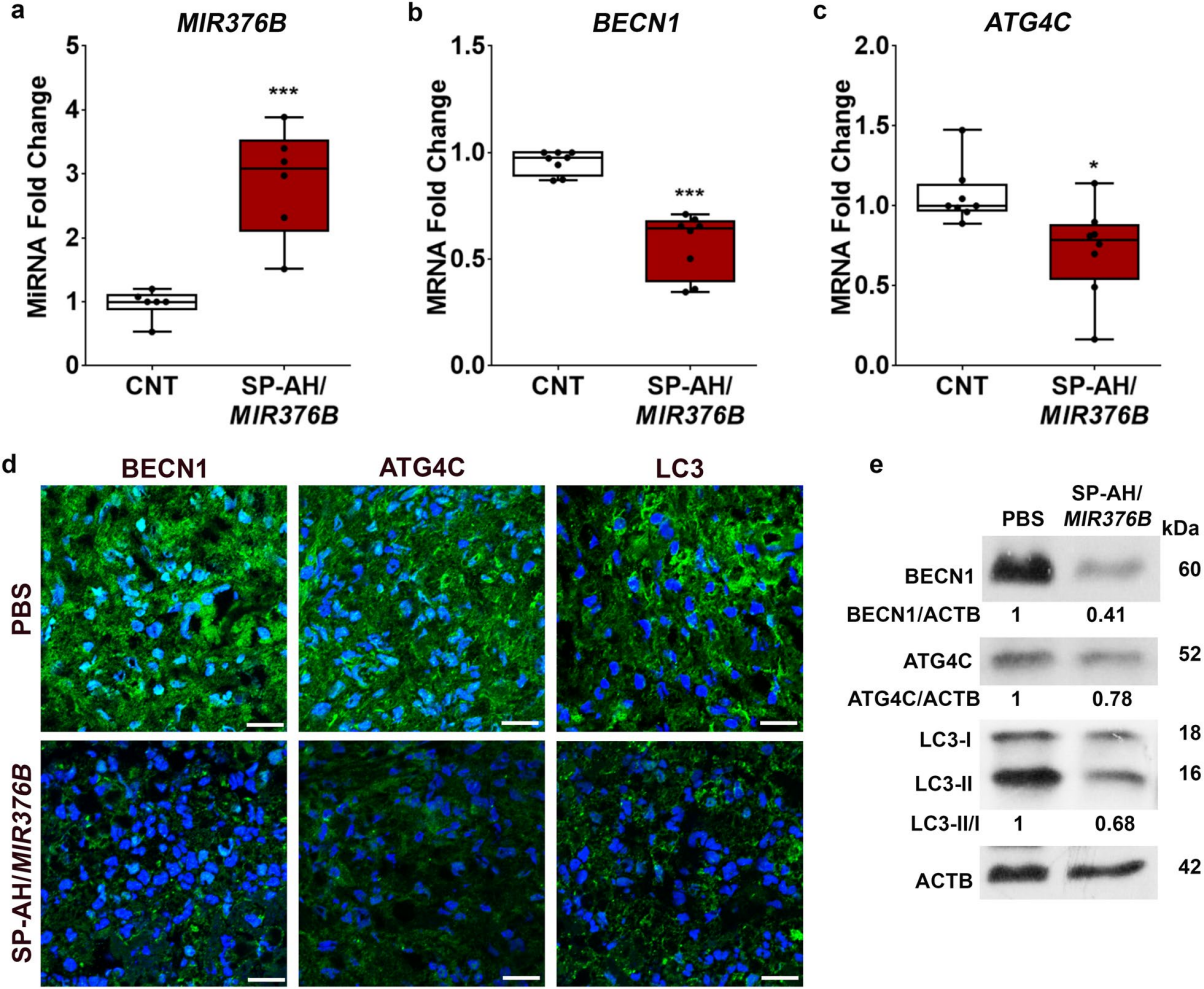
SP-AH-mediated delivery of *MIR376B* into mice blocked autophagy in xenografted breast tumors. **a***MIR376B* levels (qPCR) in xenografted MDA tumors following tail vein *i.v.* injection of PBS or SP-AH/*MIR376B* (mean ± SD, n = 6 tumors per treatment condition, ***p < 0.01). **b***BECN1* mRNA levels (qPCR) in xenografted MDA tumors (mean ± SD, n = 8 tumors per treatment condition, ***p < 0.01). **c***ATG4C* mRNA levels (qPCR) in xenografted MDA tumors (mean ± SD, n = 8 tumors per treatment condition, *p < 0.05). **d** Immunostaining of tissue sections of tumors from PBS- or SP-AH/*MIR376B*-injected mice using anti-BECN1, anti-ATG4C and anti-LC3 antibodies. **e** Immunoblot analysis of BECN1, ATG4C and LC3 in the tumor extracts from PBS- or SP-AH/*MIR376B*-injected mice. ACTB was used as loading control. BECN1/ACTB, ATG4C/ACTB and LC3-II/I band intensity ratios were marked. Scale bar: 25 µm

As shown in Fig. 6a, *MIR376B* levels were increased by several folds in tumor samples that were collected from nanoparticle-injected mice compared to PBS-injected controls. MiRNAs that were delivered by nanoparticles were highly functional since *MIR376B* targets *BECN1* (Fig. 6b) and *ATG4C* (Fig. 6c) were significantly suppressed in tumor tissues. The effect of the miRNA on target protein levels were also tested and autophagy levels were evaluated using LC3 as a marker. Immunostaining of tissue sections using specific antibodies showed a clear reduction in BECN1 and ATG4C levels (Fig. 6d) in tumor samples from SP-AH/*MIR376B*-injected mice. LC3 antibodies with a higher affinity to LC3-II were used to assess the autophagic activity of tumors. Although a strong LC3-II signal was observed in control samples, tumors from SP-AH/*MIR376B*-injected mice had a clearly lower signal (Fig. 6d). MiRNA-mediated decrease in BECN1 and ATG4C protein levels were confirmed in immunoblots of tumor protein extracts (Fig. 6e). Moreover, immunoblotting with an LC3 antibody that recognized both LC3-I and LC3-II with similar affinities showed that LC3-II/I ratios were decreased in tumors from SP-AH/*MIR376B*-injected mice, confirming autophagy inhibition in these tumors (Fig. 6e). All these results showed that miRNA mimics were delivered in vivo into tumors by SP-AH nanoparticles in a targeted manner. Resulting increase in intratumoral miRNA levels was sufficient for the downregulation of miRNA target mRNAs and proteins, and for the suppression of autophagy.

### Combination of *MIR376B*-loaded SP-AH nanoparticles with cisplatin in breast cancer treatment

Increased autophagic activity in tumors was associated with metabolic adaptation and survival of cancer cells as well as their resistance to chemotherapy [45]. Consequently, combination of chemotherapeutic agents with autophagy inhibitors was tested in a number of preclinical and clinical studies [38–40]. Having established that SP-AH-mediated delivery of *MIR376B* strongly inhibited autophagy in xenografted human breast cancer tumors, we checked whether combination of miRNA-loaded functional nanoparticles would increase the efficacy of chemotherapy.

**Fig. 7:**
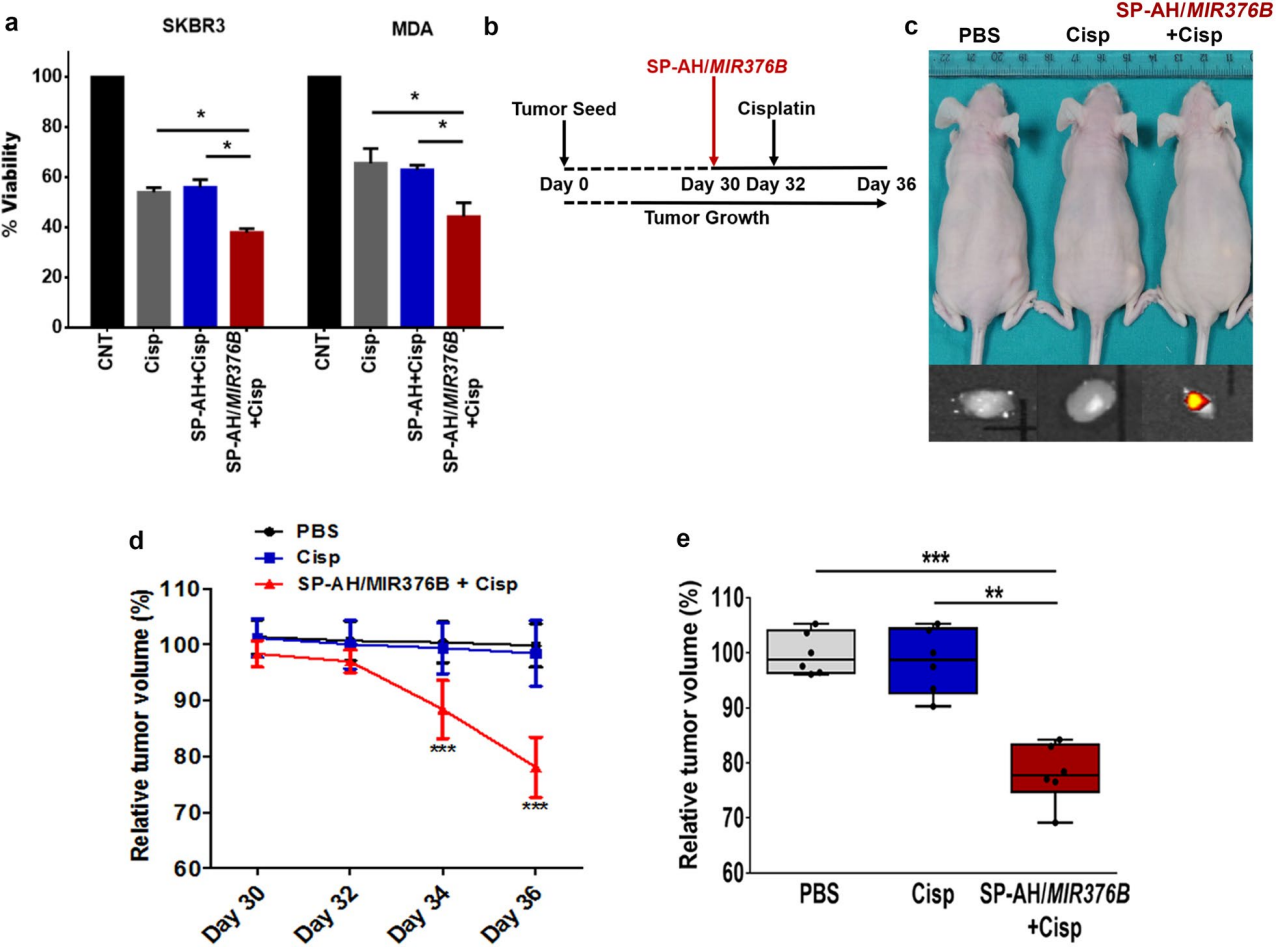
SP-AH/*MIR376B* nanoparticles potentiated cisplatin anti-cancer treatment. **a** Viability of SKBR3 and MDA cells following the treatment with PBS, Cisplatin (Cisp), SP-AH plus cisplatin or SP-AH/*MIR376B* plus cisplatin (mean ± SD of independent experiments, n = 5, *p < 0.05). **b** Scheme depicting the in vivo mice experimental set-up. 30 days after the *s.c.* injection of tumor cells, SP-AH/*MIR376B* (or PBS) were *i.v.* injected. 2 days after (Day 32), Cisplatin (or PBS) were *i.p.* injected. Tumor volumes were recorded. **c** Representative images of xenografted mice following treatments described in (**b**) and IVIS fluorescence images of isolated tumors. Scale-bars, 5 mm. **d** Time kinetics of relative changes in tumor volumes following SP-AH/*MIR376B*, Cisplatin or PBS injections into mice (Days 30–36) (mean ± SD, n = 6 tumors per condition and time point, ***p < 0.01, **p < 0.03). **e** Relative tumor volumes at Day 36 (n = 6)

First, SKBR3 or MDA cells were treated with cisplatin alone or in combination with SP-AH or SP-AH/*MIR376B* nanoparticles. While combination with SP-AH particles did not affect cisplatin toxicity in breast cancer cells, SP-AH/*MIR376B* co-treatment significantly increased anti-cancer activity of cisplatin (Fig. 7a).

Next, effects of nanoparticle-mediated autophagy inhibition on chemotherapy were tested in vivo in the xenograft mice model. Following establishment of tumors (30 days after subcutaneous injection and tumor size of 50 mm^3^), nude mice were injected with PBS, cisplatin alone or in combination with SP-AH/*MIR376B* nanoparticles. In order to clearly reveal the contribution of miRNA-loaded nanoparticles to the chemotherapeutic effect of the drug, a time point and a dose (4 days after 6 mg/kg cisplatin injection) resulting in a mild anti-tumor effect, was chosen (Fig. 7b). Under these conditions, combination of SP-AH/*MIR376B* nanoparticles with cisplatin resulted in a significant reduction in tumor volume and gave rise to superior anti-tumor effects compared to single treatment (Fig. 7c–e).

## Conclusions

One major roadblock in gene therapy is the lack of safe delivery vehicles. Nanoparticles are promising gene delivery tools, yet commonly used cationic particles are generally toxic. Based on the observation that AGO2 proteins stabilized free microRNAs in blood circulation, here we proposed the usage of AGO2 proteins as non-cationic, non-toxic and effective nucleic acid delivery agents. We demonstrated that nanoparticles functionalized by AGO2 conjugation and autophagy inhibitory miRNA addition may be used as potent adjuvant treatment tools increasing the efficacy of classical chemotherapy.

Addition of AGO2 protein as well as anti-HER2 antibody onto a PAA-coated SPION core, resulted in the creation of novel theranostic nanoparticles (SP-AH). We showed that: (i) SP-AH nanoparticles specifically and selectively targeted HER2-positive breast cancer cells and their xenograft tumors; (ii) they were efficiently internalized by cancer cells; (iii) they delivered autophagy inhibitory miRNA, *MIR376B*, into cancer cells and tumor tissues; (iv) targeted delivery of *MIR376B* successfully downregulated autophagy-related targets of the miRNA, resulting in a strong inhibition of autophagy in cells and tumors; (v) combination of *MIR376B*-loaded SP-AH nanoparticles with a classical chemotherapy drug revealed the potential of these innovative nanoparticles as adjuvant cancer treatment agents. SP-AH nanoparticles were highly biocompatible since cellular toxicity tests, blood biochemistry and complete blood count analyses, detailed histopathological examination of major organs and tissues showed no detectable short- or long-term (40 days) side-effects. All these results indicate that nucleic acid-carrier SP-AH nanoparticles have the potential to be safely used as chemotherapy enhancing novel adjuvant agents.

Mammalian AGO proteins are able to attach to various types of small RNAs with similar binding affinities [46]. Therefore, SP-AH nanoparticles may be used as general small RNA and even nucleic acid delivery agents in experimental and pre-clinical studies. Future tests, including clinical studies, will prove the potential of these particles as novel adjuvant cancer treatment agents.

## Materials and methods

### Materials

Double stranded MIR376B mimics were purchased from Dharmacon (C-300741-03-0005). FLAG-Ago2 was a gift from Edward Chan (Addgene plasmid #21538). PECFP-Rab5 and pECFP-Rab9 plasmids were kind gifts from Batu Erman. FeCl_3_·6H_2_0 and FeCl_2_·4H_2_O were purchased from Merck (U.S.A). Ammonium hydroxide (26%), poly(acrylic acid sodium salt) (Mw 2100 g/mol), Bradford reagent, bovine serum albumin (BSA) and potassium phosphate dibasic anhydrous were purchased from Aldrich (U.S.A.). Phosphate buffer saline (PBS) and 2-(*N*-morpholino) ethane sulfonic acid (MES monohydrate) buffers were purchased from Biomatik (Canada). Ethylenediaminetetraacetic acid (EDTA) disodium salt was purchased from Multicell (U.S.A.). Potassium phosphate monobasic was purchased from Riedel-de Haën (U.S.A.). *N*–*N* dimethylformamide (DMF) was purchased from Merck-Milipore (U.S.A.) ethyl-3-(3-dimethylaminopropyl) carbodiimide (EDC), *N*-hydroxysulfosuccinimide (sulfo-NHS), DyLight 650 NHS ester, Ellman’s Reagent, Traut’s Reagent and Slide-A-Lyzer 10 k and 20 k dialysis cassettes were purchased from Thermo Scientific (U.S.A.). antiHer2 was purchased from eBioscience (U.S.A.). Human AGO2 (His-tag) (EIF2C2) recombinant protein was purchased from Sino Biological Inc. (China). Maleimide-polyethyleneglycol-amine (Mal-PEG-NH2) (2 kDa) was purchased from Nanocs (U.S.A.). Vivaspin 20 (MWCO 30,000) polyethylenesulfonate (PES) filters were purchased from Sartorius (Germany). Float-A-Lyzer (MWCO 150,000) was purchased from Spectrumlabs (U.S.A).

### Characterizations

Photoluminescence spectra and absorbance spectra were (PL) recorded on a Horiba Jobin–Yvon FluoroMax-3 and UV-3600 Shimadzu UV–VIS-NIR spectrophotometer, respectively, at room temperature. Hydrodynamic size was measured by Dynamic light scattering (DLS) using Zetasizer Nano Series ZS at room temperature with 173° backscattered angle. Functional groups analysis was performed using the dried samples and Thermo Scientific Nicolet iS10 ATR-IR. Transmission electron microscopy (TEM) images were taken by Techani G2 F30 at brightfield high resolution (HR) TEM (acceleration voltage 200 kV). STEM images were taken at Hitachi HD2700 Dedicated STEM with Cs corrector (acceleration voltage 200 kV) and EDX analysis were performed with Oxford X-MaxN 100 TLE 100 mm^2^ Dual detector assignment system. Samples were deposited on carbon coated Cu-grid from dilute solutions. Vibrating Sample Magnetometry (VSM) measurements were conducted with Cryogenic limited PPMS at ambient conditions using the dried powder samples. TA Q500 (Thermogravimetric analysis (TGA)) was used to determine the organic content of SP nanoparticles under nitrogen atmosphere at a hearing rate of 10 °C/min. Tissues of nude mice models were homogenized by homogenizer, light duty, Isolab. Perkin Elmer IVIS^®^ Lumina III In Vivo Imaging System was used for optical imaging (Ex: 650, Em: 670).

### Synthesis of poly(acrylic acid) coated SPIONs (SP)

Poly(acrylic acid) **(**PAA) coated SPIONs were synthesized according to published aqueous precipitation method [47] using FeCl_3_·6H_2_O, FeCl_2_·4H_2_O, ammonium hydroxide under nitrogen. [PAA]:[Fe^2+^]:[Fe^3+^]:[OH^−^] mole ratio of 3:2:1:6 was used in the synthesis and particle growth was allowed for 1 h at 85 °C. Reaction mixture was cooled down to room temperature, placed on a 0.3 Tesla magnetic field overnight, then the precipitates were removed (if any), and colloidal suspension was washed with DI water using ultracentrifuge tubes (10 kDa MWCO PES filter). For the analysis requiring solid particles, part of the washed colloidal solution was lyophilized. The rest was stored at room temperature.

### Synthesis of AGO2 conjugated SPIONs (SP-A)

50 mg of SP was activated by mixing with 14 mg of EDC and 14.4 mg of sulfo-NHS in MES buffer at pH 6.0 and room temperature for 15 min. Then, NHS active SPIONs were washed with PBS (pH 7.4) via ultracentrifugation (10 kDa MWCO Amicon filter) at room temperature. AGO2 protein (150 µg) was added to sulfo-NHS activated nanoparticles and mixed 48 h at + 4 °C. Reaction was quenched with excess hydroxylamine and SP-A was purified with MES buffer (pH 6.0) via dialysis (150 kDa Float-A-Lyzer) for 12 h at + 4 °C with 4 times buffer refreshment.

### Activation of SP-A with maleimide groups (SP-A/MI)

SP-A was activated with EDC/sulfo-NHS and purified using the method given for SP synthesis. It was reacted with 3 mg NH_2_-PEG-Mal (2000 Da) for 48 h at + 4 °C and quenched with excess hydroxylamine. Product was purified using ultracentrifugation (10 kDa MWCO amicon filter) at + 4 °C.

### Fluorescent tagging of anti-HER2 (anti-HER2-dye)

Commercially available NHS active dye (Dylight 650^®^) was dissolved in DMF (10 mg/ml). anti-HER2 antibody was dissolved in sodium borate buffer at pH 8.55. Antibody and dye solutions were mixed in the molar ratio of [antiHer2]:[Dye] = 1:10 at room temperature for 1 h. Dye conjugated anti-HER2 was purified with dialysis using 20 kDa MWCO dialysis cassette (Slide-A-Lyzer) against phosphate/EDTA buffer (pH: 8.5) at + 4 °C with 4 times buffer refreshment in 12 h. Protein concentration and molar ratio of protein and dye was calculated using the following formula:

$$antiHer2\;Concentration\;({\text{M}}) = \frac{{\left[ {A280 - (A655 \times 0.037)} \right] \times dilution\;parameter}}{{210{,}000 \times 0.1\;{\text{cm}}}}$$

$$Dylight\;655\;per\;mol\;antiHer2\;({\text{mol}}) = \frac{A655 \times dilution\;parameter}{{73{,}000 \times protein\;concentration \times 0.1\;{\text{cm}}}}.$$

### Thiolation of anti-HER2 (anti-HER2-Dye-SH)

Anti-HER2-Dye was mixed with Traut’s Reagent at pH 8 with the molar ratio of [1:100] at room temperature for 2 h, then purified with dialysis from 10 k MWCO dialysis cassette at + 4 °C with 6 times buffer refreshment in PBS/EDTA in 4 h. Sulfhydryl groups on antiHer2 was quantified using Ellman’s reagent. For this, 25 µl Ellman’s reagent (1 mg/ml) was mixed with 25 µl of anti-HER2-dye-SH at room temperature for 15 min, then absorbance at 412 nm was measured to calculate the concentration of thiol units using the Beer Lambert equation as follows:

$$A = \in \times b \times c$$

$$0.003 = 14,150\;{\text{M}}^{ - 1} \;{\text{cm}}^{ - 1} \times 0.1\;{\text{cm}} \times c.$$

### Synthesis of anti-HER2-Dye conjugated SPIONs (SPION/AGO2/anti-HER2-dye)

Freshly prepared anti-HER2-dye-SH was mixed with maleimide functionalized nanoparticles (SP-A/MI) in PBS/EDTA at pH 7.2 for 1 h at room temperature and then overnight at + 4 °C. The product was purified with the dialysis from 300 kDa MWCO dialysis membrane at + 4 °C with 4 times buffer refreshment in PBS.

### Determination of AGO2 concentration on SPIONs by Bradford Assay

Bovine serum albumin (BSA) protein at 0, 1, 2, 4, 6, 8, 10 µg/ml in PBS was prepared as the reference. Dialysis product (dialysate) of SP-A was concentrated and measured as the analyte. 1 ml of each reference and analyte samples were mixed with 1 ml of Bradford Reagent for 10 min at room temperature, then absorbance at 595 nm was recorded using UV–vis spectroscopy. Data from the BSA was used to create the calibration curve (Adj. R^2^ = 0.9950). Since SPIONs have some absorbance at that wavelength, the amount of AGO2 was confirmed directly by the analysis of SP-A (66 µg/ml; 5.5 µg AGO2 per mg of nanoparticles) and indirectly by the analysis of unbounded AGO2 protein recovered from the dialysate. Unbounded amount of AGO2 was calculated as 11.6 µg/ml which agrees with the bounded amount.

### Determination of anti-HER2 concentration on SPIONs by photoluminescence detection

Amount of DyLight 650^®^ conjugated to anti-Her2 and SPIONs was calculated from the luminescence intensity of the dye at 672 nm. Briefly, first a calibration curve for DyLight 650^®^ was created from the dye dissolved in PBS in 2 × 10^−2^, 1 × 10^−2^, 4 × 10^−3^, 2 × 10^−3^, 1 × 10^−3^, 5 × 10^−4^ µM concentrations and their emission intensity at 672 nm was recorded on Fluoromax-3. Linear fit provided the following equation to be used in the sample analysis: PL intensity = 3.01 × 10^8^ DyLight 650^®^ conc. + 1.2 × 10^5^, R^2^ = 0.998. Emission intensity of SP–AH was measured as 1.8 M (a.u) at 672 nm (dilution factor = 20) and placed into the equation to find concentration of dye on SPIONs (0.114 nmol). To calculate the concentration of antiHer2 on SPIONs, the molar ratio between anti-HER2 and dye was used (anti-HER2:Dye = 1:0.8). According to this calculation, the mole number of antiHer2 was found as 0.142 nmol. Using the molecular weight of anti-HER2 (148,000 g/mol) concentration of anti-HER2 in SPION solution was calculated as 19 µg/ml.

This information, molecular weight of anti-HER2 (148,000 g/mol) and the mole ratio of antiHer2:Dye (1:08) indicates that dye labeled SP-AH nanoparticles contain 0.142 nmol or 19 µg/ml anti-HER2.

### Quantification of number of AGO2 and anti-HER2 per nanoparticle

Number of proteins per nanoparticle was calculated using the following equation:

$$n = a \times N \times \frac{V}{{\frac{4}{3}\pi r^{3} }}$$

where a is the mole number of proteins in 1 cm^3^, N is Avogadro’s number, V is the volume of nanoparticles in 1 cm^3^ and r is the mean radius of nanoparticles (z-average hydrodynamic size). V is calculated by subtracting volume of water in 1 cm^3^ nanoparticle suspension. Volume of water in 1 cm^3^ dispersion was also found over mass of water in 1 cm^3^ dispersion: Mass_water_ = Mass_liquid NPs_ − Mass_solid NPs_.

### Analysis of iron content in the mice tissues by inductively coupled plasma mass spectroscopy (ICP-MS) method

In order to digest the tissues and etch the iron oxide nanoparticles, the weighed organs were homogenized in the concentrated 500 μls of H_2_SO_4_, HNO_3_ and H_2_O_2_ and aged for a week. After complete digestion of the tissues, the resulting solutions were diluted to 20 ml with distilled water and the contents were detected by ICP-MS using iron standard curve as a reference, then the amount of iron per mg of organ was calculated.

### Cell culture and transfection

HEK293T human embryonic kidney cells and the breast cancer cell lines MCF7 and MDA-MB-453 were cultured in Dulbecco’s modified Eagle’s medium (DMEM, Biological Industries, #BI01-050-1A), and the other breast cancer cell line SKBR3 was cultured in McCoy’s 5A medium (PAN, #P04-05500). Both of the culture media were supplemented with 10% (v/v) fetal bovine serum (FBS, PAN, #P30-3302), antibiotics (Penicillin/streptomycin, Biological Industries, #BI03-031-1B) and l-glutamine (Biological Industries, #BI03-020-1B) in a 5% CO_2_ humidified incubator at 37 °C. HEK293T cells were transiently transfected with calcium phosphate transfection method according to standard protocols. SKBR3 cells were transfected with polyethyleneimine (PEI) method according to standard protocol.

### MiRNA loading to nanoparticles

*MIR376B* microRNAs were loaded to nanoparticles by incubating nanoparticles in an RNA binding buffer (100 mM KCl, 2 mM MgCl_2_, 10 mM Tris–HCl in DEPC water) together with 20 nM *MIR376B* mimics at 4 °C for 2 h by rotating. 150 µg/ml of each nanoparticle or equal volume of PBS with nanoparticles were incubated in the same way and cells were treated with these nanoparticles or PBS for 48 h.

### Immunoblotting and antibodies

Cells were lysed at indicated time points in RIPA buffer (50 mM TRIS–HCl pH 7.4, 150 mM NaCl, 1% NP40, 0.25% Na-deoxycholate) supplemented with a complete protease inhibitor cocktail (Roche, 04-693-131-001) and 1 mM phenylmethylsulfonyl fluoride (PMSF, Sigma-Aldrich, P7626). Protein extracts (30 µg per well) were separated using 10–15% SDS-polyacrylamide gels (SDS-PAGE), and then transferred onto nitrocellulose membranes (Millipore, #IPVH00010). Membranes were blocked in 5% nonfat milk (Applichem, #A0830) in PBST (3.2 mM Na_2_HPO_4_, 0.5 mM KH_2_PO_4_, 1.3 mM KCl, 135 mM NaCl, and 0.05% Tween 20, pH 7.4) for 1 h and then incubated with primary antibodies in a 3% BSA PBST solution: anti-AGO2 (Cell Signaling Technology, #2897), anti-Flag (Sigma, A494842), anti-BECN1 (Santa Cruz, sc-11427), anti-ATG4C (Sigma-Aldrich, AB75056), anti-LC3 (CST #2775) and anti-β-ACTIN (Sigma-Aldrich, A5441) antibody as loading control. Following PBST washes, membranes were incubated with horseradish peroxidase (HRP)-coupled secondary anti-mouse (Jackson Immunoresearch laboratories, #115035003) or anti-rabbit antibodies (Jackson Immunoresearch Laboratories, #111035144). Band intensities were quantified using the ImageJ software [48]. For dot blot assays, 96-well Bio-Dot module (BioRad, #1706547) was used. 50 µg of protein extracts were transferred onto nitrocellulose membrane under vacuum according to the manufacturer’s instructions. Then, the membrane was blocked, washed and treated with primary and secondary antibodies as described above. Full blot images of representative experiments supported in Additional file 1: Fig. S8.

### Immunofluorescence analyses

Cells were cultured on cover slides and treated with 150 µg of nanoparticles for indicated time periods, then fixed in ice-cold 4% paraformaldehyde/PBS. For colocalization experiments, cells were transfected in advance with GFP-Rab5orGFP-Rab9 plasmids and treated with nanoparticles after 48 h. Cells were fixed in ice-cold 4% paraformaldehyde/PBS. Following the fixation, nuclei were stained using Hoechst (Invitrogen, 31716 W) in PBS. Coverslides were mounted onto glass slides, and samples were analyzed with a Carl Zeiss LSM 710 confocal microscope (Zeiss, Germany). For indirect immunostaining experiments, following fixation, cells were permeabilized in PBS containing 0.1% BSA (Sigma, #A4503) and 0.1% saponin (Sigma, #84510). As primary antibodies, anti-LC3 (Sigma, L8918), anti-BECN1 (Santa Cruz, sc-11427) and anti-ATG4C (Sigma-Aldrich, AB75056) were used. Anti-mouse Alexa Fluor 488 (Invitrogen, #A32723) and anti-rabbit (Invitrogen, #A-11008) were used as secondary antibodies. Coverslides were mounted onto glass slides, and samples were analyzed using a BX60 fluorescence microscope (Olympus, BX60) or Carl Zeiss LSM 710 confocal microscope (Zeiss, Germany).

### Immunoprecipitation

HEK293T cells were cultured and transfected with flag-AGO2 for 72 h. 25 µg of anti-Flag M2 Affinity Gel beads were put into an eppendorf tube and prewashed with 1 ml cold PBS, 3 times with RIPA and 2 times with RIPA++ (supplemented with protease inhibitors cocktail and 1 mM PMSF) for 1 min at 6000 rpm, at + 4 °C. 2000 µg of protein solution was loaded on beads (Sigma, # A2220), rotated overnight at + 4 °C. After o/n incubation, beads were washed with RIPA++ and centrifuged at 6000 rpm, at + 4 °C, 1 min for 5 times. Then beads were treated with loading dye for IP control with anti-flag antibody and TRIzol reagent for RNA isolation. For long-term miRNA stability test, immunoprecipitated beads were incubated at room temperature for 21 days, and then RNA isolation was done with TRIzol reagent.

### RNA isolation and RT-PCR analyses

Total RNA was extracted using TRIzol reagent (Sigma-Aldrich, #T9424) according to the manufacturer’s instructions. cDNA was reverse transcribed from DNase-treated total RNA using M-MuLV reverse transcriptase (Fermentas, #EP0351), random hexamers (Invitrogen, #48190-011) and MIR376B stem-loop primer. For single-step qRT-PCR reaction, SYBR Green Quantitative RT-PCR kit (Roche, #04-913-914-001) and a LightCycler 480 (Roche) were used. To activate the SYBR green, an initial cycle of 95 °C, for 10 min was performed followed by PCR reactions: 40 cycles of 95 °C for 15 s and 60 °C for 1 min. Then a thermal denaturation protocol was used to generate the dissociation curves for the verification of amplification specificity (a single cycle of 95 °C for 60 s, 55 °C for 60 s and 80 cycles of 55 °C for 10 s). Changes in mRNA levels were quantified with the 2^−ΔΔCT^ method and normalized to *GAPDH* (*glyceraldehyde*-*3*-*phosphate dehydrogenase*) mRNA. Primers used during the study were: BECN1 primers 5′-AGGTTGAGAAAGGCGAGACA-3′; 5′-GCTTTTGTCCACTGCTCCTC-3′; ATG4C primers 5′-GCATAAAGGATTTCCCTCTTGA-3′; 5′-GCTGGGATCCATTTTTCG-3′, and GAPDH primers 5′-AGCCACATCGCTCAGACAC-3′; 5′-GCCCAATACGACCAAATCC-3′. TaqMan qRT-PCR reactions were performed using FastStart Universal Probe Master kit (ROCHE, #04913957001) and LightCycler 480 (Roche) according to the protocols described previously [36]. Primers and the probe used during the study were: *MIR376B*, Stem-loop primer, 5′-GTCGTATCCAGTGCAGGGTCCGAGGTATTCGCACTGGATACGACAACATGG-3′; Forward primer, 5′-GTTAATCATAGAGGAAAAT-3′; Reverse primer, 5′-GTGCAGGGTCCGAGGT-3′; TaqMan Probe, 5′(6-FAM)- GCA GGG GCC ATG CTA ATC TTC TCT GTA TCG -(TAMRA-sp)3′; U6, Forward primer, 5′-CTCGCTTCGGCAGCACA-3′; Reverse primer, 5′-RAACGCTTCACGAATTTGCGT-3′; TaqMan Probe 5′(6-FAM)-GCA GGG GCC ATG CTA ATC TTC TCT GTA TCG-(TAMRA-Sp)3′.

### Flow cytometry analysis

Nanoparticles were labelled with Alexa-flour-647 dye in order to monitor the targeting specificity of anti-Her2 tagged nanoparticles for HER2 receptor overexpressing cells via flow cytometry. MCF7, MDA-MB-453 and SKBR3 cells were seeded onto 12-well plate and treated with the nanoparticles for indicated times. At the end of incubation, cells were washed 2 times with PBS and trypsinized. Cells were fixed by using ice-cold 70% ethanol and then resuspended in PBS. The Alexa-flour-647 positive cells were detected using BD FACS Canto™ instrument and analyzed using Flow Jo software (Tree Star Inc). A minimum of 10,000 events per sample corresponding to gated population were collected, and cellular debris was not counted. Nanoparticles without an anti-HER2 antibody on their surface in which Alexa-flour-647 dye were conjugated on SPION/PAA (SP-F) and SPION/PAA/AGO2 (SP-AF) nanoparticles, were served for understanding of anti-HER2 antibody-derived targeting.

### Cytotoxicity assay

A 3-(4,5-dimethylthiazol-2-yl)-2,5-diphenyl tetrazolium bromide (MTT) assay was performed to evaluate the influence of the cisplatin, nanoparticle and the combination on cell viability. MDA-MB-453 and SKBR3 cells were seeded onto 96-well plates and cultured with the same conditions above. After 24 h, cells were treated with cisplatin or nanoparticle complexes for 48 h. Prior to the MTT reagent administration we washed the media and 10 µl of 5 mg/ml MTT reagent was added into 100 µl of cell medium and incubated for 4 h in a 5% CO_2_ humidified incubator at 37 °C. At the end of 4 h, the medium was removed, and formazan crystals were solubilized with dimethyl sulfoxide (DMSO) for 10 min at room temperature. The absorbance at 570 nm was determined with a reference filter at 650 nm for each well using a Microplate absorbance reader (Biorad, iMark). Cell viability of each group was expressed as a percentage of viability of untreated control cells.

### In vivo studies

All animal experiments were approved by the Ethics Committee of the Koç University in accordance with the guidelines on animal care and use (File No: 2013-3). NPs were passed through 0.2 micron sterile filter before administered to animals. For cytotoxicity assays, nude mice (6–8 weeks) were intravenously (*i.v.*) injected with (10 mg Fe per kg of mice) nanoparticles and inoculated for indicated time periods (10 or 40 days). At the end of inoculation, blood samples were collected by intracardiac puncture under anesthesia, and serum biochemical parameters [ALB (albumin), ALKP ((alkaline phosphatase), ALT (alanine aminotransferase), AMYL (amylase), Ca (calcium), GLU (glucose), LIPA (lipase), TBIL (total bilirubin), TP (total protein), BUN (blood urea nitrogen) and GLOB (globulin)] were analyzed by using diagnostic health profile kit (IDEXX VetTestTM, #6802590) on an Olympus AU400 according to manufacturer’s instructions. Complete blood count test was performed with the same mice on machine (PROKAN, #PE-6800).

Formalin-fixed mice tissues embedded in paraffin, cut at 4-micron using a microtome. After transfer on slides and air-drying and they were baked for 15 min at 60 °C. Deparaffinization and rehydration were achieved by immersing slides in xylene, 100% ethanol, and 70% ethanol. Following the boiling in 10 mM sodium citrate buffer (pH 6.0), and cooling, slides were counterstained with hematoxylin and eosin.

A xenograft mouse model was generated by subcutaneous injection of SKBR3 and MDA-MB-453 cells [1 × 10^7^ cells per mouse embedded in Matrigel (BD Biosciences, #356234)] in the right front flank of female nude mice (6–8 weeks, ~ 24 g). When the tumors reached ~ 50–100 mm^3^, approximately 4 weeks after inoculation, the tumor bearing mice were injected via tail vein a single dose of nanoparticles (10 mg/kg Fe^+2^). At the indicated time points, in vivo imaging of the nanoparticles was conducted under isoflurane inhalation anesthesia by using IVIS Spectrum imaging system (Caliper Life Sciences) with the Living Image software package. Anesthetized mice were euthanized through cervical dislocation and dissected to remove tumors, livers, brain, intestine, stomach, uterus, kidneys, spleens, hearts, and lungs which were placed into 10% Formalin for pathology investigation.

For microRNA delivery experiments, MIR376B microRNAs were bound to SP-AH nanoparticles as explained above and *i.v.* injected to mice bearing MDA-MB-453 tumor. After 6 days of injection, mice were euthanized, and RNA and protein levels were investigated. For cisplatin-combination analysis, tumors were generated, and nanoparticle were injected as the above. Then, 2 days after the nanoparticle injection, 6 mg/kg cisplatin were intraperitoneally (*i.p.*) injected. 6 mg/kg dose is equivalent to the human equivalent dose used in the clinic [49] and in line with previous animal studies [50, 51]. Four days after cisplatin injection, relative changes in tumor volume were determined by caliper measurements and compared to measurements in PBS injected control mice.

### Statistical analyses

Statistical analyses were performed using Student’s two-tailed *t* test. Data were represented as means of ± SD of independent experiments (biological replicates). Values of p < 0.05 were considered as being significant (*p < 0.05, **p < 0.03, ***p < 0.01). Reactions were performed in duplicates and number of independent experiments (n) was marked.

## Supplementary information

**Additional file 1: Figure S1.** AGO2 protein binding improved *MIR376B* were abilized. (a) Overexpression of flag-AGO2 protein in HEK293T cells for 48 h and immunoprecipitation efficiency determined by pre- and flag-IP using flag antibody. p3Xflag was used as control transfection. (b) Comparative Taqman qPCR analysis of immunoprecipitated amount of *MIR376B* by RNA isolation from flag beads incubated with cell lysate of HEK293T cells with or without flag-AGO2 overexpression. (c) Determination of *MIR376B* level on flag-beads after 21 days of flag-AGO2 immunoprecipitation at room temperature by RNA isolation and Taqman qPCR. Taqman qPCR data was normalized using *U6 small nuclear 1(RNU6-1)* (U6) mRNA levels. **Figure S2.** Characterization of nanoparticles. (a) Zeta potential of SP-AH in PBS buffer (pH: 7.4) (b) Powder XRD pattern provided with the peak assignments of SP. (c) Thermogram of SP and PAA. (d) FTIR spectra of bare SPION, pure PAA and SP. **Figure S3.** (a) Measurement of AGO2 concentration of SP-A by Bradford assay. Standard curve generated from the absorbance of BSA at 595 nm as a function of BSA concentration (µg/mL). (b) Photoluminescence (PL) spectra of Dylight 650® at decreasing concentrations (2 × 10^−2^), 1 × 10^−2^, 4 × 10^−2^, 2 × 10^−3^, 1 × 10^−3^, 5 × 10^−4^ µM, λex: 655 nm, inset: Calibration curve of PL intensity vs concentration of DyLight 650®. PL spectra of (c) SP-AH (d) SP-F and (e) SP-AF. (λex: 655 nm, dilution factor: 20). **Figure S4.** Biocompatibility of SP and SP-AH nanoparticles *in vitro* and *in vivo* systems. (a) Determination of viability of 3 different breast cancer cells, MCF7, SKBR3 and MDA-MB-453, treated with increasing concentrations (5-500 µg/ml) of SP or SP-AH nanoparticles for 48 h by MTT cell viability assay. (b) Body weight change after 10 and 40 days in mice *i.v.* injected with SP, SP-AH (10 mg Fe per kg of mice) or equal volume of PBS. (c) Hematoxylin and eosin staining of mice tissues after 40 days of PBS, SP and SP-AH injections. **Figure S5.** Uptake and biodistribution of SP-AH nanoparticles in mice. (a) Iron amount in different mice tissues and tumor measured by Inductively Coupled Plasma (ICP) analysis one day after *i.v.* injection of PBS or SP-AH (10 mg Fe per kg of mice). (b) *Ex vivo* IVIS images of organs after one day of PBS or SP-AH *i.v.* injections. **Figure S6.** Determination of SP-AH nanoparticles on microRNA level *in vitro*. (a, b) QPCR analysis of SP-AH (150 µg/ml) treatment on *MIR376B* and its targets, *BECN1* and *ATG4C*, after 48 h of treatment in SKBR3 (a) and MDA-MB-453 (b) cells. Control (CNT) cells were treated with equal amount of PBS with SP-AH treatment. QPCR data was normalized using *U6 small nuclear 1 (RNU6-1)* mRNA for *MIR376B* and *GAPDH* for *ATG4C* and *BECN1* mRNAs. (mean ± SD of independent experiments, n = 3, *p < 0.05). **Figure S7.** Characterization of synthesized nanoparticles. (a) STEM micrograph of SP (b) Elemental analysis of SP showing the Fe and O (Scale bar: 20 nm). **Figure S8.** Full blot images of representative experiments that were presented in the manuscript. (a-e) Corresponding Figure numbers were marked. Processed areas were shown in a rectangle. **Figure S9.** HER2 status analysis by anti-HER2 antibody staining of MCF-7 and MDA cells. (a) Graphic demonstration and (b) FACS quadrant of HER2 positivity by FACScan. (c) Confocal imaging of SP-AH (150 µg/ml) treated MCF-7 and MDA cells. Scale bar: 25 µm. **Figure S10.** Comparative analysis of different aged SP-AH NPs. (a) Freshly prepared, (b) 6 month aged, (c) 1 year aged NPs analyzed. Lower part: Confocal imaging of targeting capacity; Upper part: QPCR analysis of *MIR376B* target *BECN1* level after 48 h of treatment of SP-AH (150 µg/ml) in MDA-MB-453 cells. Scale bar: 25 µm. **Figure S11.** SP-AH/*MIR376B* nanoparticles anti-cancer effect on breast cancer cell lines. Viability of SKBR3 and MDA cells following the treatment with SP-AH, Cisplatin or SP-AH/*MIR376B* (n = 3, n.s.; not significant).

## Data Availability

All data and material are included in the article and its additional files.
