# Supplementary material for: Treatment of breast cancer with autophagy inhibitory microRNAs carried by AGO2-conjugated nanoparticles
Source: J Nanobiotechnology. 2020 Apr 28;18:65. doi: 10.1186/s12951-020-00615-4 (PMC7189576; doi:10.1186/s12951-020-00615-4)
Supplement: Supplementary file 1 — Additional file 1: Figure S1. AGO2 protein binding improved MIR376B were abilized. (a) Overexpression of flag-AGO2 protein in HEK293T cells for 48 h and immunoprecipitation efficiency determined by pre- and flag-IP using flag antibody. p3Xflag was used as control transfection. (b) Comparative Taqman qPCR analysis of immunoprecipitated amount of MIR376B by RNA isolation from flag beads incubated with cell lysate of HEK293T cells with or without flag-AGO2 overexpression. (c) Determination of MIR376B level on flag-beads after 21 days of flag-AGO2 immunoprecipitation at room temperature by RNA isolation and Taqman qPCR. Taqman qPCR data was normalized using U6 small nuclear 1(RNU6-1) (U6) mRNA levels. Figure S2. Characterization of nanoparticles. (a) Zeta potential of SP-AH in PBS buffer (pH: 7.4) (b) Powder XRD pattern provided with the peak assignments of SP. (c) Thermogram of SP and PAA. (d) FTIR spectra of bare SPION, pure PAA and SP. Figure S3. (a) Measurement of AGO2 concentration of SP-A by Bradford assay. Standard curve generated from the absorbance of BSA at 595 nm as a function of BSA concentration (µg/mL). (b) Photoluminescence (PL) spectra of Dylight 650® at decreasing concentrations (2 × 10^−2^), 1 × 10^−2^, 4 × 10^−2^, 2 × 10^−3^, 1 × 10^−3^, 5 × 10^−4^ µM, λex: 655 nm, inset: Calibration curve of PL intensity vs concentration of DyLight 650®. PL spectra of (c) SP-AH (d) SP-F and (e) SP-AF. (λex: 655 nm, dilution factor: 20). Figure S4. Biocompatibility of SP and SP-AH nanoparticles in vitro and in vivo systems. (a) Determination of viability of 3 different breast cancer cells, MCF7, SKBR3 and MDA-MB-453, treated with increasing concentrations (5-500 µg/ml) of SP or SP-AH nanoparticles for 48 h by MTT cell viability assay. (b) Body weight change after 10 and 40 days in mice i.v. injected with SP, SP-AH (10 mg Fe per kg of mice) or equal volume of PBS. (c) Hematoxylin and eosin staining of mice tissues after 40 days of PBS, SP and SP-AH injections. Figure S5. Uptake an [file 12951_2020_615_MOESM1_ESM.docx]

**Figure S1.** AGO2 protein binding improved *MIR376B* were abilized.

**Figure S2.** Characterization of nanoparticles.

**
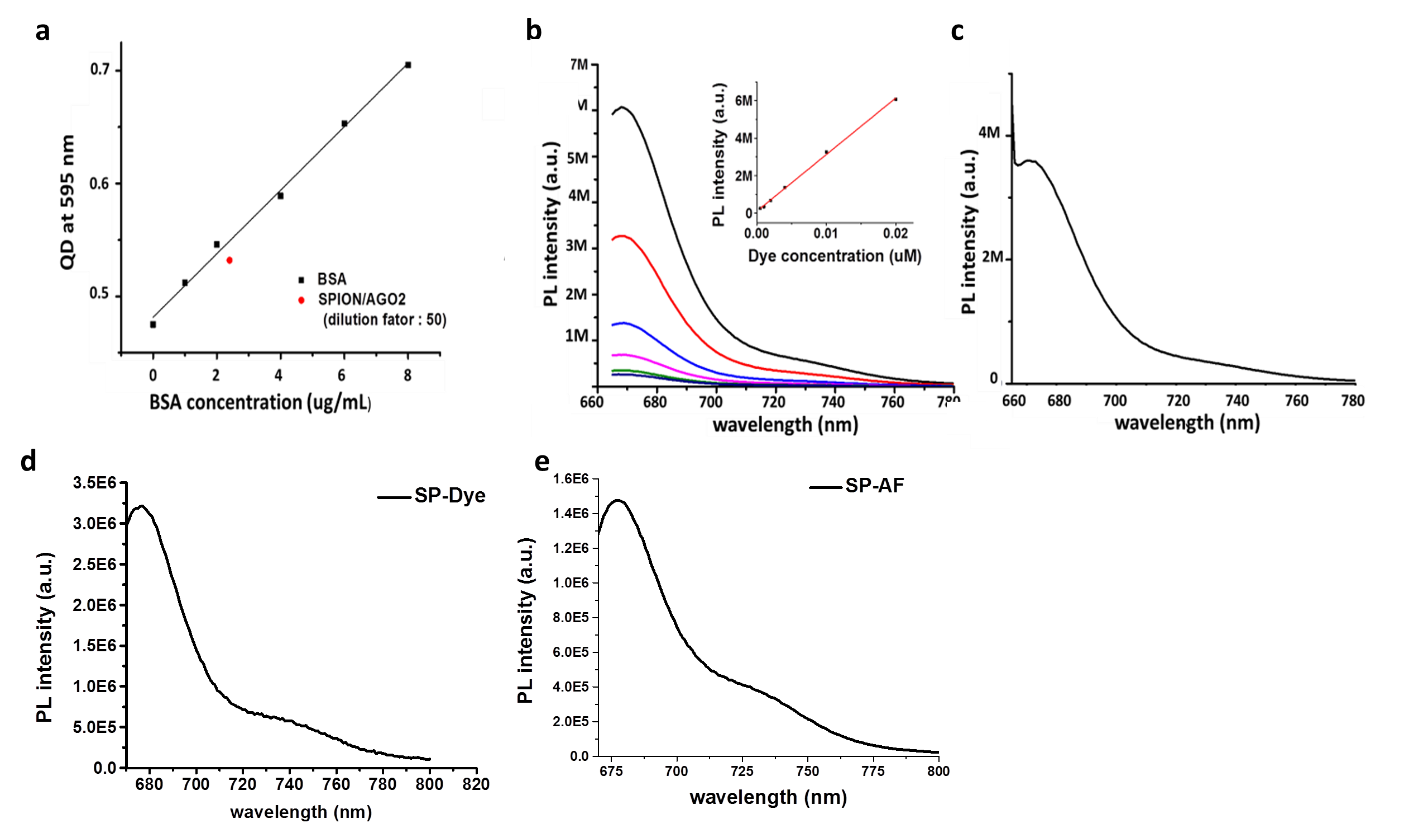
**

**Figure S3.** Characterization of nanoparticles.

**
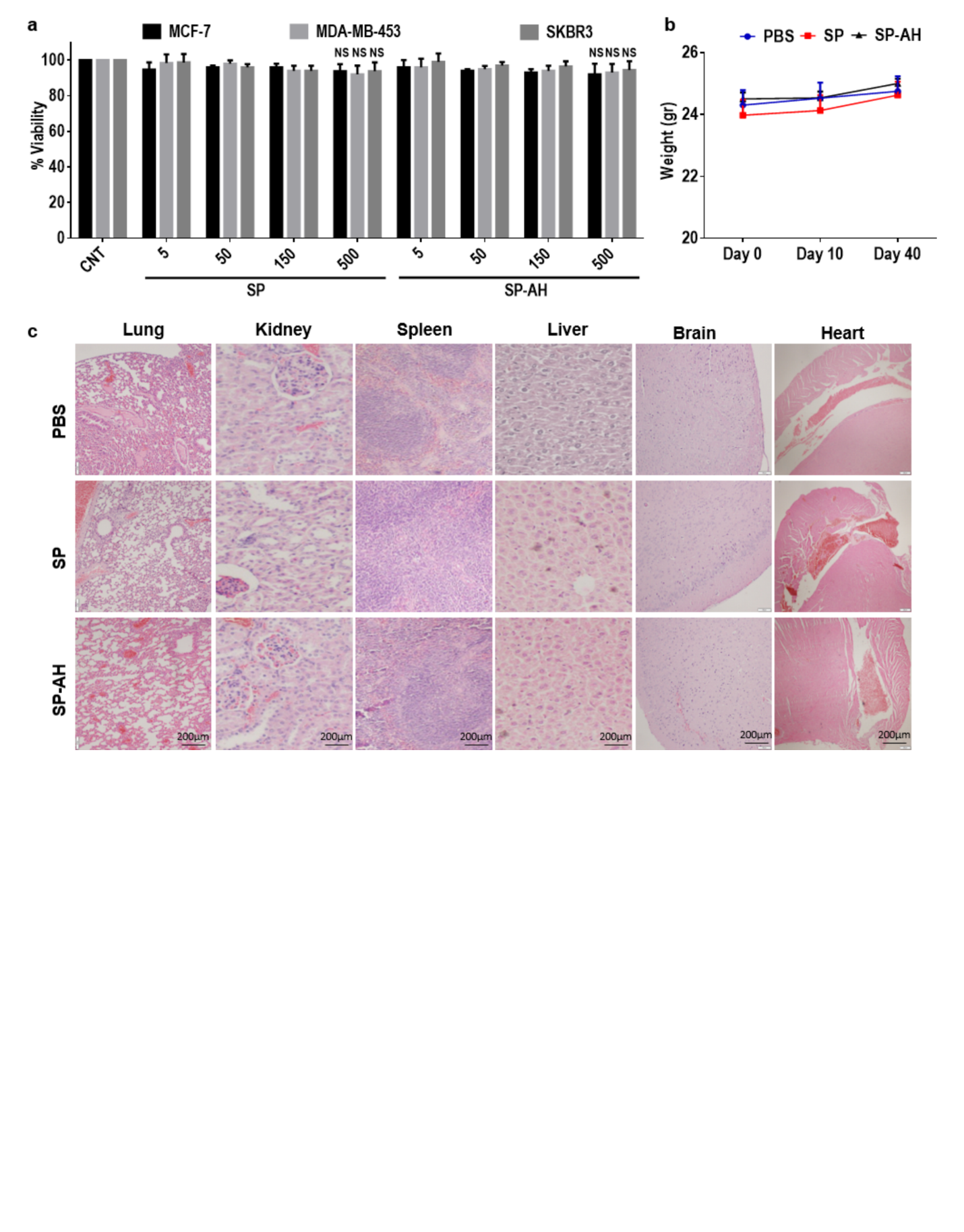
**

**Figure S4.** Biocompatibility of SP and SP-AH nanoparticles *in vitro* and *in vivo* systems.

**
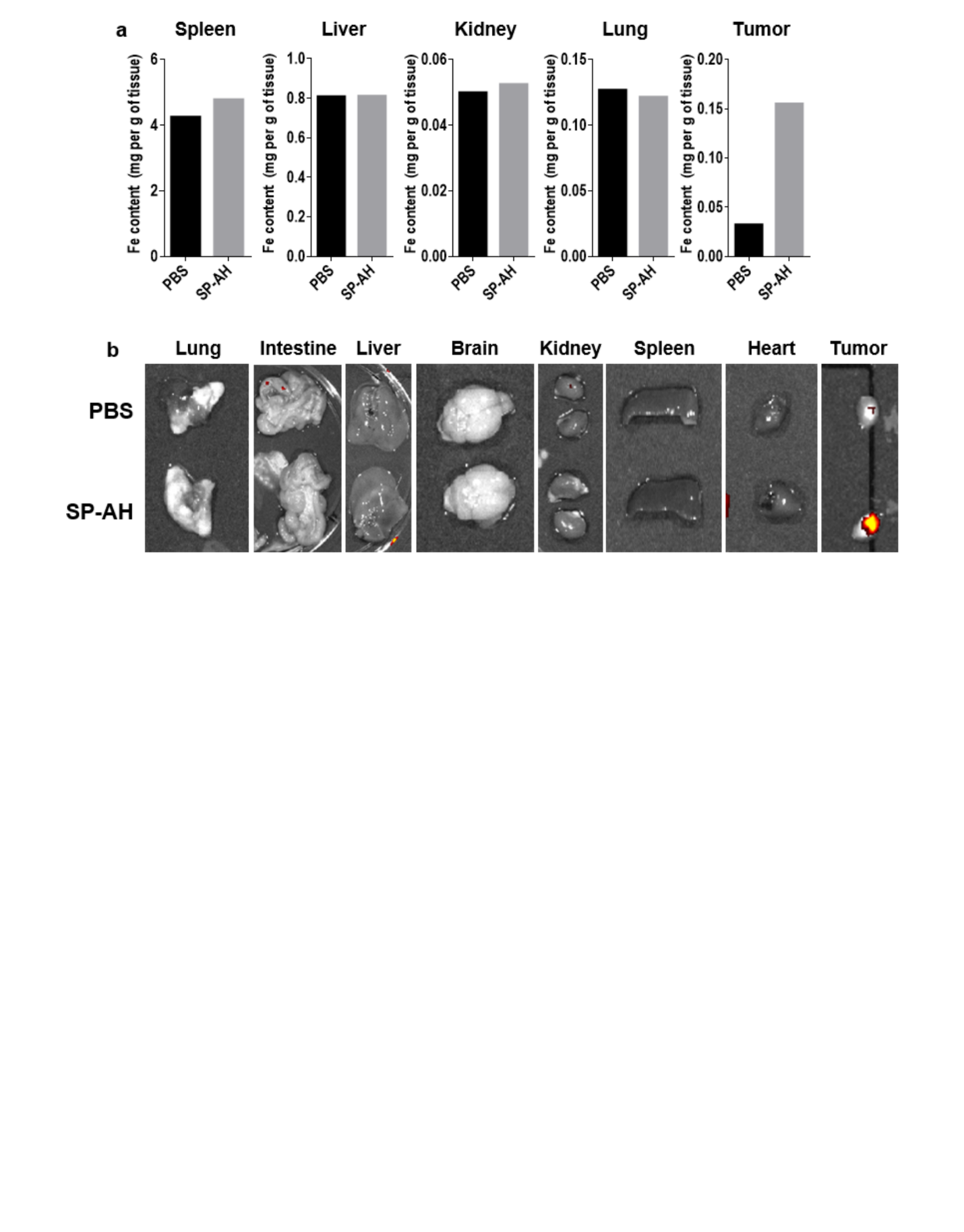
**

**Figure S5.** Uptake and biodistribution of SP-AH nanoparticles in mice.

**
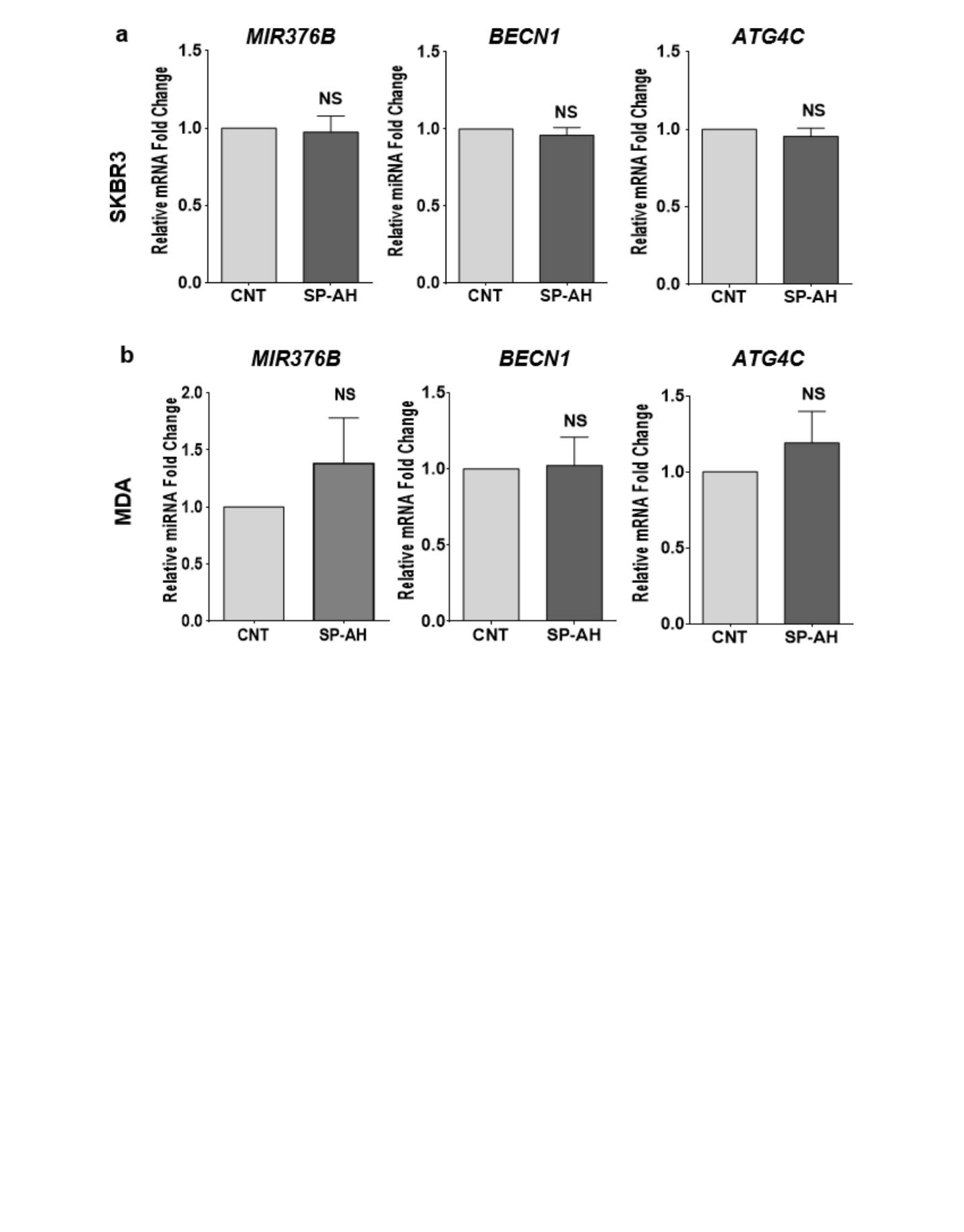
**

**Figure S6.** Determination of SP-AH nanoparticles on microRNA level *in vitro*.

**
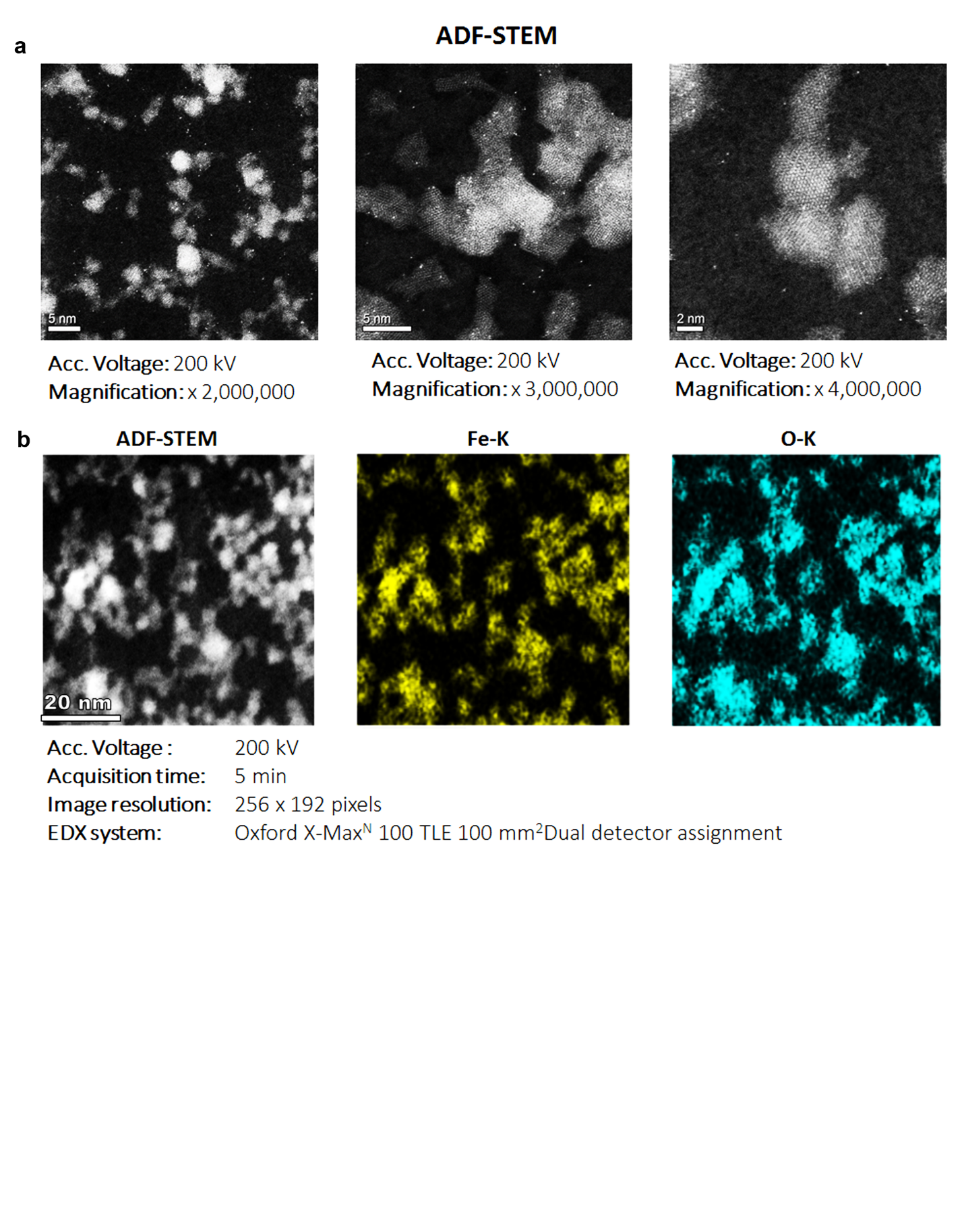
**

**Figure S7.** Characterization of synthesized nanoparticles.

**Figure S8.** Full blot images of representative experiments that were presented in the manuscript.


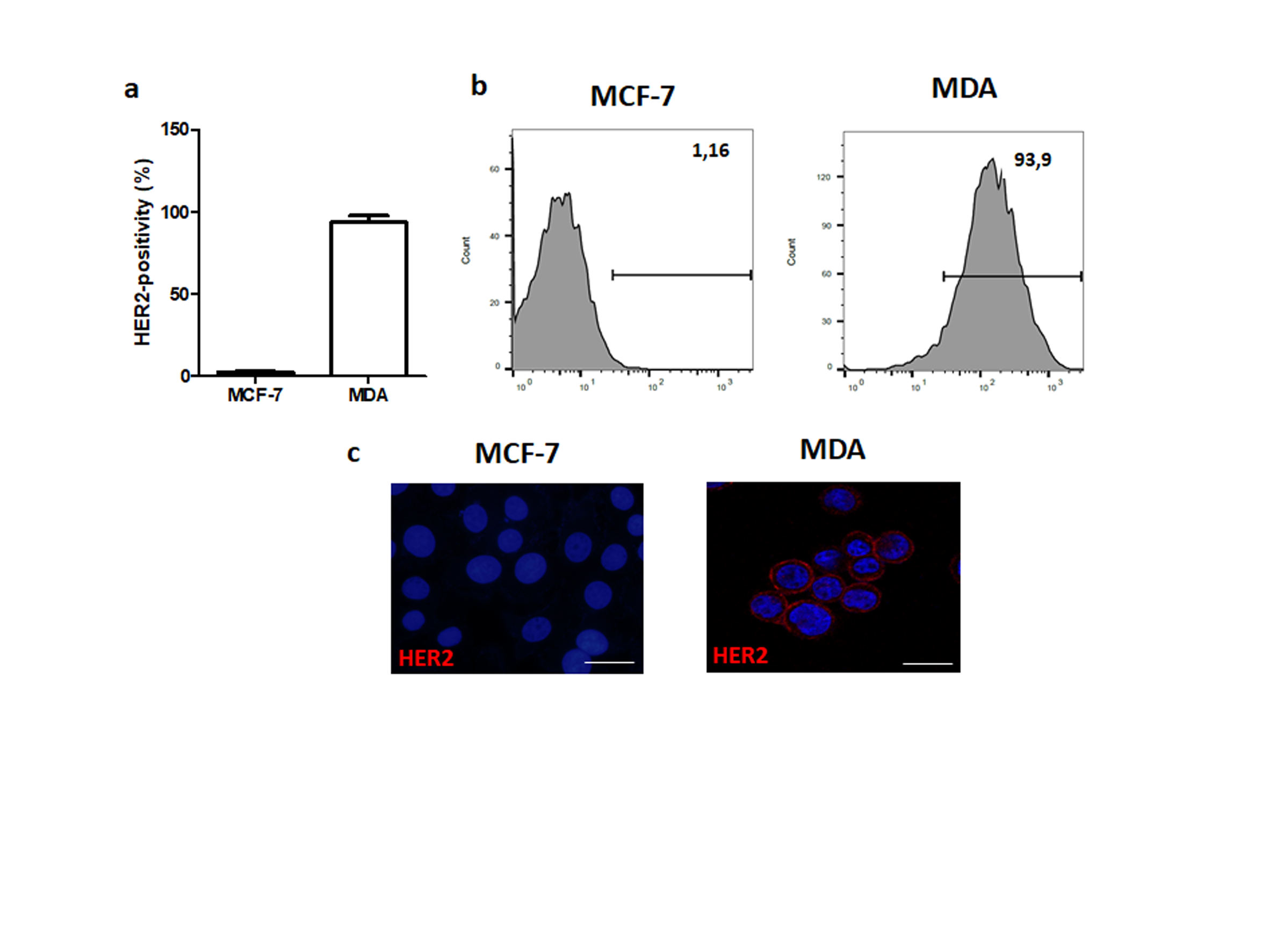


**Figure S9.** HER2 status analysis by anti-HER2 antibody staining of MCF-7 and MDA cells. (a) Graphic demonstration and (b) FACS quadrant of HER2 positivity by FACScan. (c) Confocal imaging of SP-AH (150 µg/ml) treated MCF-7 and MDA cells.


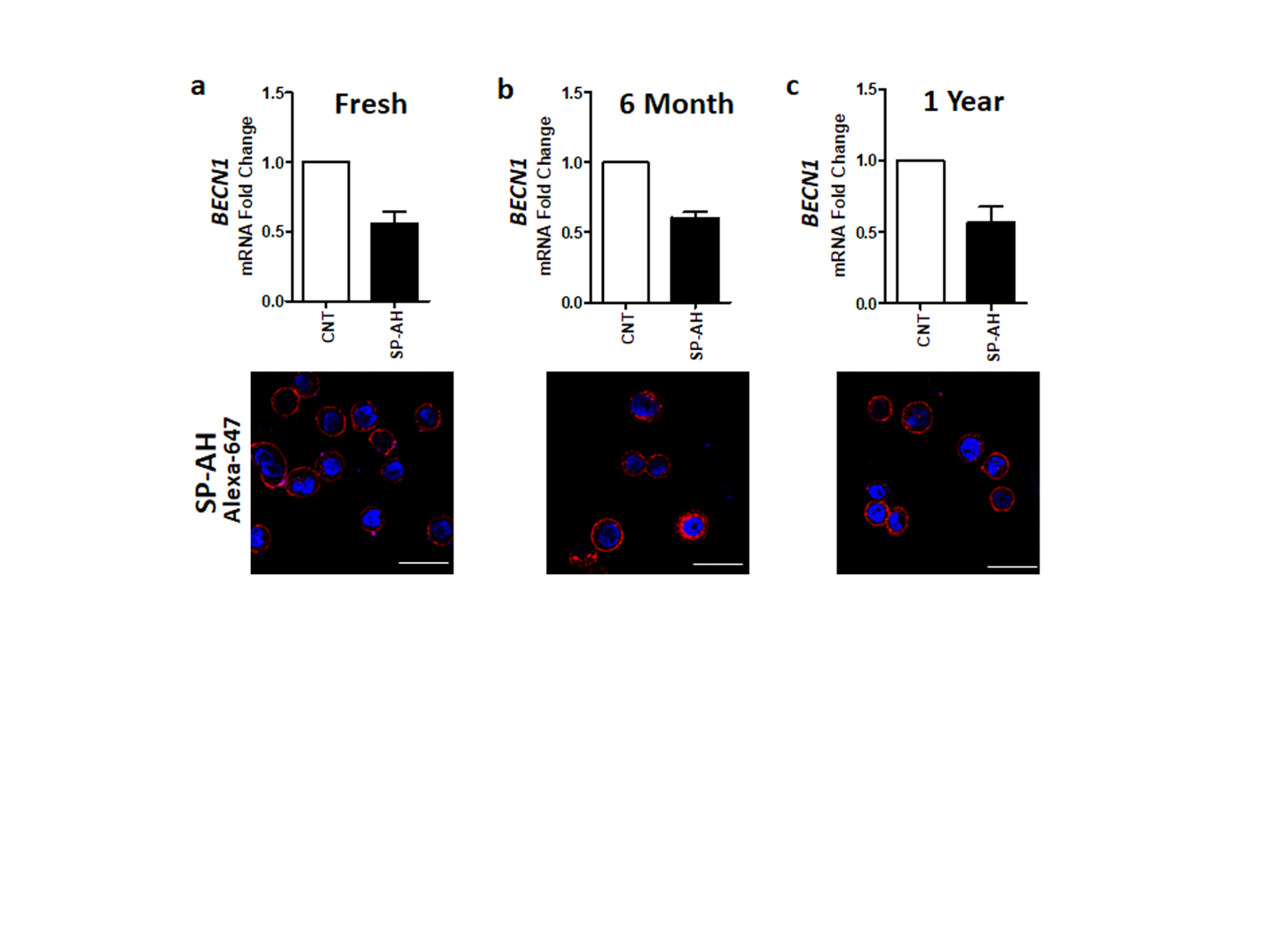


**Figure S10.** Comparative analysis of different aged SP-AH NPs. (a) Freshly prepared, (b) 6 month aged, (c) 1 year aged NPs analyzed. Lower part: Confocal imaging of targeting capacity; Upper part: QPCR analysis of *MIR376B* target *BECN1* level after 48 hours of treatment of SP-AH (150 µg/ml) in MDA-MB-453 cells.





**Figure S11.** SP-AH/*MIR376B* nanoparticles anti-cancer effect on breast cancer cell lines. Viability of SKBR3 and MDA cells following the treatment with SP-AH, Cisplatin or SP-AH/*MIR376B* (n=3, n.s.; not significant).

Table S1: Blood biochemistry analysis of nanoparticle injected nude mice

|  | **Normal Range** | **PBS** | **SP**  **10 days** | **SP-AH**  **10 days** | **SP**  **40 days** | **SP-AH**  **40 days** |
| --- | --- | --- | --- | --- | --- | --- |
| **ALB** | 2.5-4.8 | 2.4 | 2.4 | 2.4 | 2.4 | 2.3 |
| **TBIL** | 0.1-0.9 | 0.2 | 0.2 | 0.2 | 0.2 | 0.2 |
| **ALKP** | 62-209 | 96 | 75 | 82.5 | 113 | 81 |
| **ALT** | 28-132 | 67 | 81 | 67.5 | 66 | 70 |
| **Ca** | 5.9-9.4 | 10.7 | 9.1 | 9.05 | 10 | 10.3 |
| **BUN** | 18-29 | 21 | 20 | 23 | 21 | 21 |
| **GLU** | 90-192 | 204 | 235 | 184 | 210 | 243 |
| **LIPA** | U/L | 777 | 819 | 806 | 724 | 734 |
| **AMYL** | 1691-3615 | 2253 | 2074 | 1862.5 | 2094 | 1828 |

Table S2: Complete blood count

|  | **PBS** | **SP**  **10 days** | **SP-AH**  **10 days** | **SP**  **40 days** | **SP-AH**  **40 days** |
| --- | --- | --- | --- | --- | --- |
| **WBC** | 16.8 | 1.3 | 29 | 27.3 | 54.1 |
| **LYM%** | 47.7 | 51.8 | 49.2 | 67.9 | 79.1 |
| **MID%** | 8.1 | 5.3 | 8.9 | 7.3 | 6.3 |
| **GRAN%** | 44.2 | 42.9 | 41.9 | 24.8 | 14.6 |
| **LYM#** | 8 | 0.7 | 14.3 | 18.5 | 42.8 |
| **MID#** | 1.4 | 0.1 | 2.6 | 2 | 3.4 |
| **GRAN#** | 7.4 | 0.5 | 12.1 | 6.8 | 7.9 |
| **RBC** | 8.21 | 5.5 | 7.66 | 8.86 | 8.1 |
| **HGB** | 16.7 | 9.8 | 15.9 | 18.2 | 15.8 |
| **HCT** | 44.6 | 30.8 | 40.2 | 50.1 | 44.3 |
| **MCV** | 54.4 | 56.1 | 52.6 | 56.6 | 54.8 |
| **MCH** | 20.3 | 17.8 | 20.7 | 20.5 | 19.5 |
| **MCHC** | 37.4 | 31.8 | 39.5 | 36.3 | 35.6 |
| **RDW-SD** | 24.1 | 24.1 | 24.1 | 26 | 27.9 |
| **RDW-CV** | 14.2 | 14 | 14.6 | 14.9 | 16.2 |
| **PLT** | 1350 | 154 | 758 | 1163 | 998 |
| **MPV** | 9.9 | 10.1 | 11.2 | 9.8 | 10.5 |
| **PDW** | 10 | 9.2 | 7.7 | 9.7 | 10 |
| **PCT** | 1.33 | 0.15 | 0.84 | 1.13 | 1.04 |
| **P-LCR** | 21.4 | 19.9 | 26.2 | 18.8 | 24.9 |
